# *FLOWERING LOCUS T4* delays flowering and decreases floret fertility in barley

**DOI:** 10.1093/jxb/eraa466

**Published:** 2020-10-13

**Authors:** Rebecca Pieper, Filipa Tomé, Artem Pankin, Maria von Korff

**Affiliations:** 1 Institute for Plant Genetics, Heinrich Heine University Düsseldorf, Düsseldorf, Germany; 2 Max Planck Institute for Plant Breeding Research, Cologne, Germany; 3 Cluster of Excellence on Plant Sciences, ‘SMART Plants for Tomorrow’s Needs’, Heinrich Heine University Düsseldorf, Düsseldorf, Germany; 4 University of Nottingham, UK

**Keywords:** Barley, cereals, fertility, flowering, FLOWERING LOCUS T, photoperiod

## Abstract

*FLOWERING LOCUS T*-like (*FT*-like) genes control the photoperiodic regulation of flowering in many angiosperm plants. The family of *FT*-like genes is characterized by extensive gene duplication and subsequent diversification of *FT* functions which occurred independently in modern angiosperm lineages. In barley, there are 12 known *FT*-like genes (*HvFT*), but the function of most of them remains uncharacterized. This study aimed to characterize the role of HvFT4 in flowering time control and development in barley. The overexpression of *HvFT4* in the spring cultivar Golden Promise delayed flowering time under long-day conditions. Microscopic dissection of the shoot apical meristem revealed that overexpression of *HvFT4* specifically delayed spikelet initiation and reduced the number of spikelet primordia and grains per spike. Furthermore, ectopic overexpression of *HvFT4* was associated with floret abortion and with the down-regulation of the barley MADS-box genes *VRN-H1*, *HvBM3*, and *HvBM8* which promote floral development. This suggests that HvFT4 functions as a repressor of reproductive development in barley. Unraveling the genetic basis of *FT*-like genes can contribute to the identification of novel breeding targets to modify reproductive development and thereby spike morphology and grain yield.

## Teaser

We identify the *FLOWERING LOCUS T* (*FT*)-like gene *HvFT4* as a negative regulator of reproductive development, spikelet initiation, floret fertility, and grain number in barley.

## Introduction

Variation in flowering time was crucial for the successful adaptation of crop plants to many different geographic areas and strongly impacts yield and reproductive success (Turner *et al.*, 2005; Cockram *et al.*, 2007; Verhoeven *et al.*, 2008; Comadran *et al.*, 2012; Campoli and von Korff, 2014; Gol *et al.*, 2017).

Flowering time is a complex trait regulated by environmental (photoperiod, ambient temperature, and vernalization) and internal cues (autonomous, circadian clock, age, gibberellin, and sugar availability) (Mouradov *et al.*, 2002; Fornara *et al.*, 2010; Andrés and Coupland, 2012). In Arabidopsis, the different endogenous and environmental cues are integrated by the key floral integrator *FLOWERING LOCUS T* (*FT*) (Kardailsky *et al.*, 1999; Kobayashi *et al.*, 1999). *FT* is expressed under long-day (LD) conditions in the leaves and then translocated as a protein to the shoot apical meristem (SAM), where it interacts with the basic leucine zipper (bZIP) transcription factor FLOWERING LOCUS D (FD) to activate the expression of meristem identity genes to induce floral transition (Abe *et al.*, 2005; Wigge *et al.*, 2005; Corbesier *et al.*, 2007). Large *FT*-like gene families have been found in the genomes of cereal monocots such as wheat and barley (12 FT paralogs each), rice (13 FT paralogs), and maize (15 FT paralogs) (Chardon and Damerval, 2005; Faure *et al.*, 2007; Danilevskaya *et al.*, 2008; Halliwell *et al.*, 2016). Several studies have demonstrated that these *FT*-like gene families arose from gene duplication events followed by subfunctionalization or neofunctionalization within and between species. One example of subfunctionalization of FT-like paralogs can be found in perennial poplar trees (*Popolus* spp.), where *FT1* and *FLOWERING LOCUS T2* (*FT2*) have functionally diverged to coordinate flowering and growth cycles (Hsu *et al.*, 2011). Furthermore, functional diversification has also been demonstrated in rice, where two FT1-like paralogs, *HEADING DATE3a* (*Hd3a*) and *Rice FT1* (*RFT1*), act as photoperiod-specific florigens. *Hd3a* is induced under inductive short-day (SD) conditions to promote flowering, in contrast to its closest homolog *RFT1* which acts as major floral activator under LD conditions (Kojima *et al.*, 2002; Hayama *et al.*, 2003; Tamaki *et al.*, 2007; Komiya *et al.*, 2008). Similarly, in barley, *FT1* and *FT2* are expressed under LDs and promote floral development, while *FT3* is expressed under SDs and LDs and induces spikelet initiation (Digel *et al.*, 2015; Mulki *et al.*, 2018; Shaw *et al.*, 2019). However, the multifaceted roles of *FT*-like genes after extensive gene duplication events within most flowering species have not yet been described.

Barley is a facultative LD plant with either a winter or a spring growth habit. Growth habit and vernalization requirement are determined by the genetic interaction of *VERNALIZATION 1* (*VRN-H1*) and *VERNALIZATION 2* (*VRN-H2*). *VRN-H2* encodes a zinc finger and CCT domain- [for CONSTANS (CO), CONSTANS-LIKE (CO-like), and TIMING OF CAB EXPRESSION1 (TOC1)] containing protein and is expressed under LDs before winter (Yan *et al.*, 2004). The *APETALA1* (*AP1*)/*CAULIFLOWER* (*CAL*)/*FRUITFULL* (*FUL*)-like MADS box transcription factor gene *VRN-H1* is a repressor of *VRN-H2* and its up-regulation during vernalization releases *HvFT1* and *HvFT3* expression (Yan *et al.*, 2003, 2004; Mulki *et al.*, 2018). Allelic variation in *VRN-H1* (deletions in the regulatory regions of the first intron) and *VRN-H2* (deletion of the gene locus) induce a vernalization-independent expression of *HvFT1*, resulting in a spring growth habit (Hemming *et al.*, 2009; Rollins *et al.*, 2013). Photoperiod response is controlled by *PHOTOPERIOD 1* (*PPD-H1*), a homolog of the Arabidopsis PSEUDO RESPONSE REGULATOR proteins with a pseudo-receiver and a CCT domain (Turner *et al.*, 2005). Under inductive day length (LDs), *PPD-H1* activates *HvFT1* transcription in the leaves and thereby accelerates flowering (Laurie *et al.*, 1995; Turner *et al.*, 2005; Yan *et al.*, 2006; Hemming *et al.*, 2008). Spring barley varieties carry a mutation in the CCT domain of *Ppd-H1* that is associated with a decreased *HvFT1* expression level under LD conditions and a delay in flowering (Turner *et al.*, 2005; Hemming *et al.*, 2008). While *HvFT1* is only expressed and induces flowering under LD conditions, the homolog *HvFT3* is expressed and promotes reproductive development under LD and SD conditions (Mulki *et al.*, 2018). Experiments in wheat, barley, *Brachypodium*, and rice suggested that *FT2* is a floral promoter, downstream of *FT1*, and expressed in the leaf as well as in the inflorescence (Kikuchi *et al.*, 2009; Lv *et al.*, 2014; Digel *et al.*, 2015; Shaw *et al.*, 2019).

The role of the barley *FT* paralogs *HvFT4*–*HvFT12* is as yet undescribed, and there is no information on their function in barley or the related species wheat. Previous studies have demonstrated that *FT*-like genes modulate different developmental traits and processes. A better understanding of the function of *FT-*like genes in cereals is therefore important to optimize their development and performance. This study aimed to functionally characterize the role of *HvFT4* in flowering time and reproductive development in barley. Specific goals were to characterize the effects of *HvFT4* overexpression on macroscopic and microscopic inflorescence development under LDs and study the pleiotropic effect of *HvFT4* overexpression on vegetative and reproductive traits. Another objective was to identify target genes of *HvFT4* by analyzing the expression of the main flowering time genes in the leaves and inflorescence in response to *HvFT4* overexpression. Finally, the amino acid sequence of HvFT4 was compared with that of several known floral repressors and promoters from diverse species to identify common amino acid changes in conserved motifs related to flowering time control. This study demonstrates that overexpression of *HvFT4* delayed flowering specifically by delaying spikelet initiation, and negatively affected fertility, and grain and tiller number.

## Materials and methods

### Generation of transgenic *Ubi::HvFT4* lines

Transgenic *Ubi::HvFT4* lines were generated as described for *Ubi::HvCO1* (Campoli *et al.*, 2012a), *Ubi::HvCO2* (Mulki *et al.*, 2016), and *Ubi::HvFT3* (Mulki *et al.*, 2018). The *HvFT4* fragment was cloned from cDNA (cv. Optic) and is identical to the Morex sequence DQ411320.

### Plant material and growth conditions

Three independent transgenic T_1_ and T_2_ families designated *Ubi::HvFT4* lines 491 (OX-491), 483 (OX-483), and 517 (OX-517) were sown in 96-well growing trays (Einheitserde, 100 ml per cell) together with a null segregant line (null) and the wild-type spring cultivar Golden Promise (WT). The null segregant sister line without the transgene was used as a control together with Golden Promise. Golden Promise is a spring barley with a mutation in the CCT domain of *Ppd-H1*, a deletion of the vernalization gene *VRN-H2*, and a deletion in the first regulatory intron of the vernalization gene *VRN-H1* (Mulki *et al.*, 2018; Gol *et al.*, 2021). Golden Promise therefore does not need vernalization to flower and is characterized by a reduced induction of the *HvFT1* and late flowering under LD conditions. The grains were stratified at 4 °C for 3 d for even germination and then transferred to the greenhouse (LDs, 16 h light/8 h darkness) and controlled temperatures (20 °C/16 °C days/nights). Germination was recorded as the day of coleoptile emergence from the soil and the plants were transferred to single pots (Einheitserde, 1 liter per pot) 14 days after emergence (DAE). Repotted plants were randomized following a random block design.

### Genotyping by PCR

To confirm the *Ubi::HvFT4* insertion, genomic DNA was extracted following the Biosprint DNA extraction protocol (Qiagen) and was eluted in 200 µl of deionized water. *Ubi::HvFT4* plants were screened for the presence of the transgene using primers that amplify the hygromycin selectable marker gene (Vec8_F/Vec8_R), located on the transformation vector, and the HvFT4 cDNA sequence (FT4_tg_1F/nos_tg_1R), but not the *HvFT4* genomic DNA (see Supplementary Table S1 at *JXB* online). Amplifications were performed in 1× Green GoTaq Reaction Buffer (Promega) with 5 µl of 1:3 diluted template DNA, 0.5 U of GoTaq G2 DNA Polymerase (Promega), 64 µM PCR Nucleotide Mix (Promega), 1.5 mM MgCl_2_, and 0.2 µM each of the upstream and downstream primer. Conditions were as follows: 95 °C (3 min), 34 cycles of 95 °C (30 s), 57.5 °C (1 min), 72 °C (1 min), and 72 °C (10 min). A 12 µl aliquot of the PCR product was visualized on a 2% agarose gel with ethidium bromide as dye.

### Confirmation of *HvFT4* overexpression in transgenic *Ubi::HvFT4* lines

To confirm *HvFT4* overexpression, the middle part of the youngest fully emerged leaf of main shoots was collected 12 DAE 10 h after lights-on (Zeitgeber time T10). Samples were immediately frozen in liquid nitrogen and stored at –80°C until RNA extraction and real-time quantitative reverse transcription–PCR (qRT–PCR) as described below.

### Phenotyping

Phenotypes were recorded for 6–32 plants of each of the transgenic lines, the null segregant, and Golden Promise. Flowering time was measured in days from emergence until heading date. Heading was scored as the appearance of 1 cm of the awns from the main shoot flag leaf sheath (Zadoks stage 49) (Zadoks *et al.*, 1974). Morphological phenotypes of the shoot were recorded at heading such as the number of tillers, number of leaves on the main culm, leaf size, and plant height, or at plant maturity such as peduncle extrusion. The leaf size (width and length) of the flag leaf and of the three youngest leaves before the flag leaf (Leaf A, B, and C) was measured on the main culm. The leaf width was measured at the widest point of the leaf blade, and the leaf length was measured from the ligule to the tip. The height of the plants was measured as the distance from the crown to the collar of the main shoot flag leaf. Peduncle extrusion of the main shoot was measured as the distance from the collar of the flag leaf to the base of the spike.

Plants were harvested individually and the total number of florets on the main spike as well as the number of grains on each rachis node of the main spike were recorded. Floret fertility was calculated as the relative number of grains compared with the total number of central florets. In addition, the number of fertile tillers (fertile=at least one grain) was counted. Tiller fertility was calculated as the relative number of fertile tillers compared with the total number of tillers. Total shoot dry mass and fertile spike dry mass were measured to calculate the harvest index (HI) as follows, HI=fertile spikes dry mass (g)/total shoot dry mass (g). The grains from the main shoot were cleaned, and grain width, length, and area, and thousand grain weight (TGW) were measured with the MARVIN Seed Analyser (GTA Sensorik).

### Microscopic inflorescence development and gene expression during plant development

One *Ubi::HvFT4* line (OX-517), the null segregant control, and the wild-type Golden Promise (WT) were cultivated and genotyped as described above. Three primary shoots per genotype were dissected with a microsurgical knife (5 mm blade, Surgical Specialties Corporation) under the stereo microscope every 7 d starting at 21 DAE. The development of the meristem was scored according to the quantitative Waddington scale (Waddington *et al.*, 1983). A stereo microscope (Nikon SMZ18) equipped with a digital camera (Nikon digital sight DS-U3) was used to obtain images of apices. For apex sampling, the surrounding leaves were removed from the main shoot apex and the apex was cut from the stem with the microsurgical knife. Meristematic tissue was collected at Waddington stages 3.5–4.5 and 6.0–7.0 by pooling five meristems and was immediately frozen in liquid nitrogen. In addition, a leaf sample from the selected plants was taken before dissection, as described above, and pooled in the same way as the meristems. All samples were taken at Zeitgeber time T10.

### RNA extraction, cDNA synthesis, and qRT–PCR

Leaf and meristem material for gene expression analysis was ground and subjected to RNA extraction using TRIzol reagent (Invitrogen) according to the manufacturer’s instructions with subsequent DNase I treatment (Thermo Scientific). First-strand cDNA synthesis was performed using ~4 µg of total RNA with 0.5 mM dNTP Mix (Thermo Scientific), 1 µg of oligo(dT)12–18 primer (Metabion International AG), 0.01 M DTT (Invitrogen), and 150 U of SuperScript® II Reverse Transcriptase (Invitrogen) in 1× First-Strand Buffer (final volume 40 µl) following the manufacturer’s instructions, and subsequently diluted 1:4 in nuclease-free water. qRT–PCRs were performed on cDNA samples using gene-specific primers (see Supplementary Table S1) as described in Campoli *et al.* (2012a) and Bi *et al.* (2019). Two technical replicates were used for each sample, and non-template controls were included. Starting concentrations of the target transcripts were calculated according to the absolute quantification method, with primer efficiency correction based on the titration curve for each target gene using the LightCycler 480 Software (Roche; version 1.5) and normalized against the geometric mean of the reference genes *HvActin*, *HvGAPDH*, and *HvADP* (Supplementary Table S1).

### Multiple protein alignments

To evaluate variation of the FT4 proteins in different monocot species, we identified the FT4 orthologs in the monocot genomes deposited at Ensemble Plants (Bolser *et al.*, 2017) using blastp with the HvFT4 protein sequence as a query (Altschul *et al.*, 1990). The hits with >80% identify over 90% of the HvFT4 length were extracted as the FT4 orthologs (Supplementary Table S2). The multiple alignment and annotation of the monocot FT4 proteins were performed using AliView v. 1.26 (Larsson, 2014).

To identify amino acid residues putatively involved in the antagonistic functions of *FT*-like genes, we aligned amino acid sequences from functionally characterized *FT*-like genes. Amino acid sequences from selected FT homologs from Arabidopsis, onion (*Allium cepa*), sugar beet (*Beta vulgaris*), longan (*Dimocarpus longan*), soybean (*Glycine max*), sunflower (*Helianthus annuus*), tobacco (*Nicotiana tabacum*), sugarcane (*Saccharum* spp.), and Norway spruce (*Picea abies*) were selected, which have been extensively studied in Wickland and Hanzawa (2015). This selection was expanded with FT homologs from rice (*Oryza sativa*) and wheat (*Triticum aestivum*), the FT function of which has been characterized, and all barley FT homologs (Halliwell *et al.*, 2016). Multiple protein alignments of full coding regions of PEBP (phosphatidylethanolamine-binding protein) containing FT-like proteins were created using ClustalW with default settings on the MUSCLE homepage (Multiple Sequence Comparison by Log-Expectation, https://www.ebi.ac.uk/Tools/msa/muscle/). The protein sequences were sorted into seven groups according to function and/or species. FT and TFL1 proteins from Arabidopsis were considered as group 1 and used as a reference. FT-like proteins from other cereal species with close homology to barley FT-like proteins, as determined in a phylogenetic analysis by Halliwell *et al.* (2016), were collected in group 2. Group 2 FT-like proteins are described as floral promoters and were included to confirm the conservation of the amino acid pattern, which is critical to determine FT-like function in cereal monocots. Furthermore, barley FT-like proteins with described inductive (group 3) or repressive function on flowering time (group 4), as well as FT-like proteins from various species with described function as floral promoters (group 6) or floral repressors (group 7) (Wickland and Hanzawa, 2015) were arranged accordingly in the alignment. All remaining barley *FT*-like genes with unknown function were included in group 5. Corresponding protein IDs or gene model information are listed in Supplementary Table S3.

### Statistical analysis

Data were visualized and analysed using the R software (version 1.0.153, R Development Core Team, 2008). Significant differences in flowering time, developmental stage of the SAM, morphological phenotypes, and gene expression levels between each of the *Ubi::HvFT4* families, the null control, and the WT Golden Promise were identified by one-way ANOVA followed by Tukey’s multiple comparison test (Tukey HSD) with *P*-value adjustment. The statistical significance of differences in *HvFT4* expression levels between the two phenotypic categories of *Ubi::HvFT4* (category I and 0) was determined using a Welch two-sample *t*-test. The significance level was α=0.05 for all tests.

### Diversity analysis of the *HvFT4* locus

The homozygous single nucleotide polymorphism (SNP) genotypes of the *HvFT4* locus (DQ411320) were extracted from the targeted enrichment re-sequencing data of the wild and domesticated barley diversity panel (Pankin *et al.*, 2018). The missing genotypes were partially imputed using the k-nearest neighbor genotype imputation algorithm LinkImpute (LD-kNNi) implemented in TASSEL 5.2.61 with default settings, except for the ‘Max distance between the sites to find LD’ set at 40 (Bradbury *et al.*, 2007; Money *et al.*, 2015). The formats of the genotype matrix were converted using AliView 1.26 (Larsson 2014). The median joining haplotype network of the HvFT4 haplotypes filtered by <0.05 missing positions was calculated using popart 1.7 (Bandelt *et al.*, 1999; Leigh and Bryant, 2015).

## Results

### Overexpression of *HvFT4* prolongs the vegetative phase and delays flowering

**Fig. 1.**
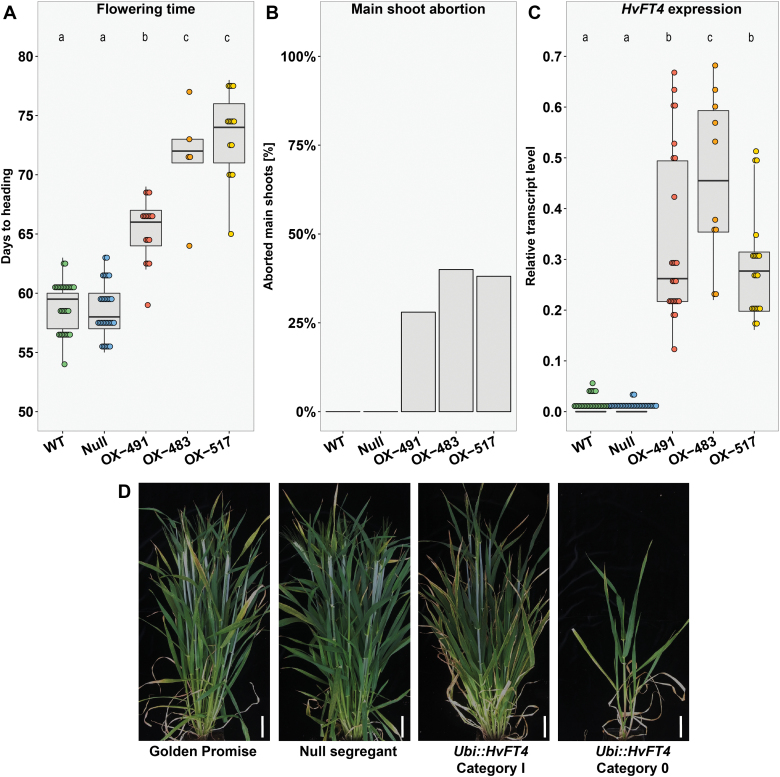
Overexpression of *HvFT4* delays flowering and leads to premature main shoot abortion. (A) Flowering time was measured in days from emergence until heading of the main shoot (Zadoks stage 49) (Zadoks *et al.*, 1974). (B) Proportion of prematurely aborted main shoots [%]. (C) *HvFT4* expression was measured in the youngest fully emerged leaf of the main shoot 12 DAE at Zeitgeber time T10 under LDs (16 h light/8 h night). The expression level of each gene was normalized against the geometric mean of the reference genes *HvActin*, *HvGAPDH*, and *HvADP.* Each dot represents the values obtained from a single plant. Statistical differences (*P*≤0.05) between families were calculated by one-way ANOVA followed by Tukey’s multiple comparison test (Tukey HSD). (D) Representative plants of every genotype. *UBI::HvFT4* plants showed two distinct phenotypes (Category I and Category 0). Category 0 plants showed impaired development and higher levels of *HvFT4* expression compared with Category I plants. Scale bar=5 cm. WT, Golden Promise; Null, null segregant, OX-491, *Ubi::HvFT4*-491; OX-483, *Ubi::HvFT4*-483; OX-517, *Ubi::HvFT4*-517.

The constitutive overexpression of *HvFT4* in the background of Golden Promise significantly delayed time to flowering (Fig. 1A). The three independent transgenic *Ubi::HvFT4* families flowered on average 71 DAE, while Golden Promise and the null segregants required on average only 59 d to flowering. Furthermore, 35% of transgenic *Ubi::HvFT4* plants exhibited an impaired main shoot development (Fig. 1B, D). These plants, herein referred to as category 0, remained small and the main shoot apex was aborted during late stem elongation and failed to flower, while the remaining 65% of the transgenic lines (category I) eventually flowered and formed a spike (Fig. 1B, D). *HvFT4* was strongly up-regulated in the transgenic lines compared with the control lines (Fig. 1C). The expression of *HvFT4* was 2-fold higher in the leaves of category 0 plants (data not shown) compared with the expression levels of *HvFT4* in plants of category 1 (*P*=5.16×10^–6^, Welch two-sample *t*-test). Thus, up-regulation of *HvFT4* expression led to a delay in flowering or even the abortion of the main shoot.

We then examined the effects of *HvFT4* overexpression on individual phases of pre-anthesis development. For this purpose, developing primary shoots of one transgenic family (OX-517) and both control lines (WT and null) were dissected and inflorescence development was evaluated according to the Waddington scale (Waddington *et al.*, 1983). This scale describes the development of the inflorescence and the most advanced floret primordium and carpel within the inflorescence. The development of the first spikelet primordia on the shoot apex at the double ridge stage (W1.5–W2.0) marks the transitions to a reproductive SAM. The first floral organ primordia differentiate at the stamen primordium stage (W3.5), when stem elongation also initiates. Anthesis and pollination of the most advanced floret take place at the Waddington stage W10.0.

**Fig. 2.**
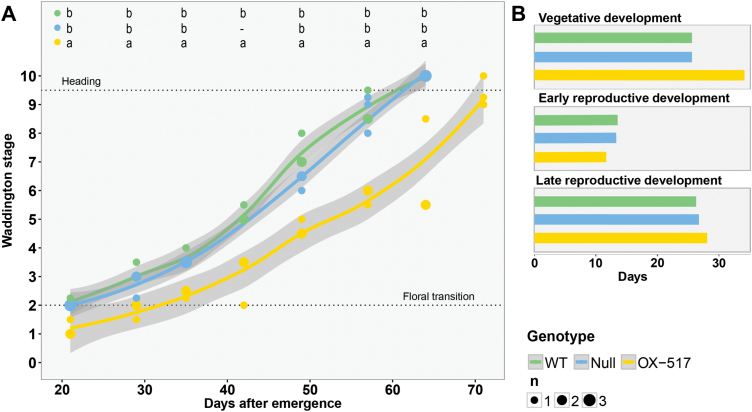
Overexpression of *HvFT4* delays development of the main shoot apex. (A) Microscopic development of the main shoot apex was scored every 7 d according to the Waddington scale (Waddington *et al.*, 1983). Three plants per genotype were dissected at each time point. Polynomial regression models at a 95% confidence interval (Loess smooth line) are shown. Statistical differences (*P*≤0.05) at each time point were calculated by one-way ANOVA followed by Tukey’s multiple comparison test (Tukey HSD). (B) Durations of three distinct developmental phases were extracted graphically from (A). The length of the vegetative phase was measured as the days from germination until Waddington stage 2.0, the early reproductive phase was accounted for as the time from Waddington stage 2.0 until 3.5, and the time from Waddington stage 3.5 until heading was considered as the late reproductive phase. WT, Golden Promise; Null, null segregant; OX-517, *Ubi::HvFT4*-517.

Apical meristems of the control plants had already initiated spikelet primordia (W2.0) 21 DAE, whereas the transgenic plants required 8 d more to reach the same stage (Fig. 2). Stem elongation (W3.5) was initiated in the transgenic lines 42 DAE, whereas the control lines had reached the same stage 7 d earlier. Golden Promise lines flowered at 64 DAE, while the transgenic line flowered only at 71 DAE (Fig. 2A). Consequently, the delay in flowering time was primarily due to a prolonged vegetative phase in the *Ubi::HvFT4* plants (Fig. 2B).

**Fig. 3.**
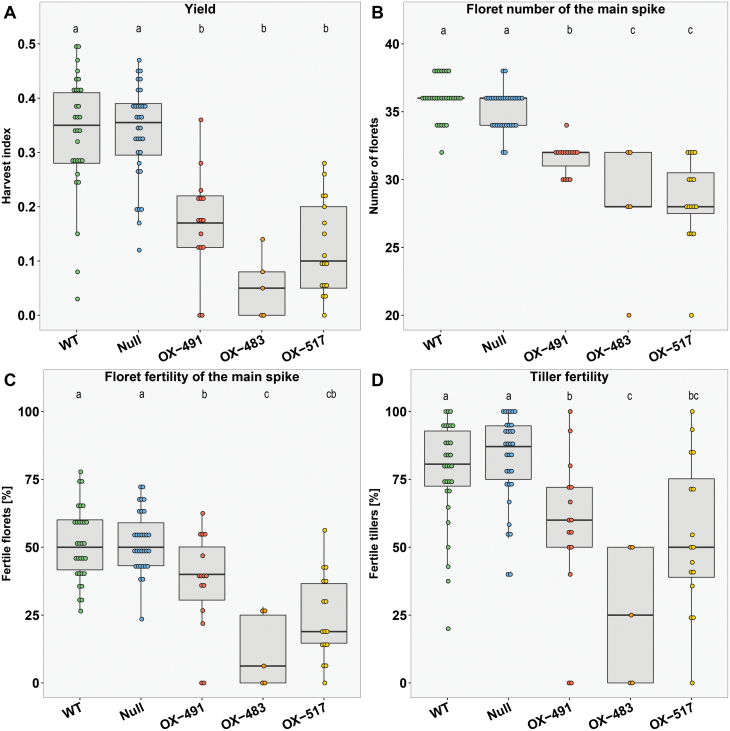
Overexpression of *HvFT4* reduces yield, tiller number, and fertility. (A) Harvest index [HI=fertile spikes dry mass (g)/total shoot dry mass (g), (B) total number of florets on the main spike, (C) number of tillers, and (D) fertile tillers [%] (=spike with at least one grain) were recorded at plant maturity. Each dot represents the values obtained from a single plant. Statistical differences (*P*≤0.05) between genotypes were calculated by one-way ANOVA followed by Tukey’s multiple comparison test (Tukey HSD). WT, Golden Promise; Null, null segregant; OX-491, *Ubi::HvFT4*-491; OX-483, *Ubi::HvFT4*-483; OX-517, *Ubi::HvFT4*-517.

The delay in reproductive development in Ubi::*HvFT4* was correlated with a reduced HI, determined as the weight of the filled spikes in proportion to the total shoot biomass of the plant (Fig. 3A). Null segregants and Golden Promise had a HI of, on average, 0.34, whereas *Ubi::HvFT4* plants had a HI of 0.17 (OX-491), 0.05 (OX-483), and 0.1 (OX-517), which corresponds to a reduction in HI of 49–84% (Fig. 3A). This reduction in HI was mainly caused by a decrease in reproductive biomass. The number of florets and the number of grains on the main spike were significantly reduced in transgenic plants compared with the control plants (Fig. 3B; Supplementary Fig. S1C). The reduction in overall spike fertility was mainly attributed to reduced grain set in the central zone of the spike in *Ubi::HvFT4* plants, whereas the apical and basal part was also partly sterile in control plants (Supplementary Fig. S2). Interestingly, *Ubi::HvFT4* plants developed significantly fewer tillers at flowering compared with control plants even though they initiated spikelet primordia significantly later and consequently formed more leaves on the main culm than the WT (Fig. 3C; Supplementary Fig. S1D). Additionally, the proportion of tillers with spikes containing at least one grain was significantly lower in *Ubi::HvFT4* compared with control plants (Fig. 3D). However, *HvFT4* overexpression had no effects on plant height, peduncle extrusion, or leaf size in this study (Supplementary Figs S1A, B, S3). Finally, TGW, grain width, and grain size were reduced in *Ubi::HvFT4* compared with the controls (Supplementary Fig. S4). Taken together, overexpression of *HvFT4* decreased reproductive biomass by reducing tiller and spike number, grain number per spike, and grain size.

### Overexpression of *HvFT4* reduced expression of the *AP1*-like flowering promoters in the leaves and the shoot apical meristem

**Fig. 4.**
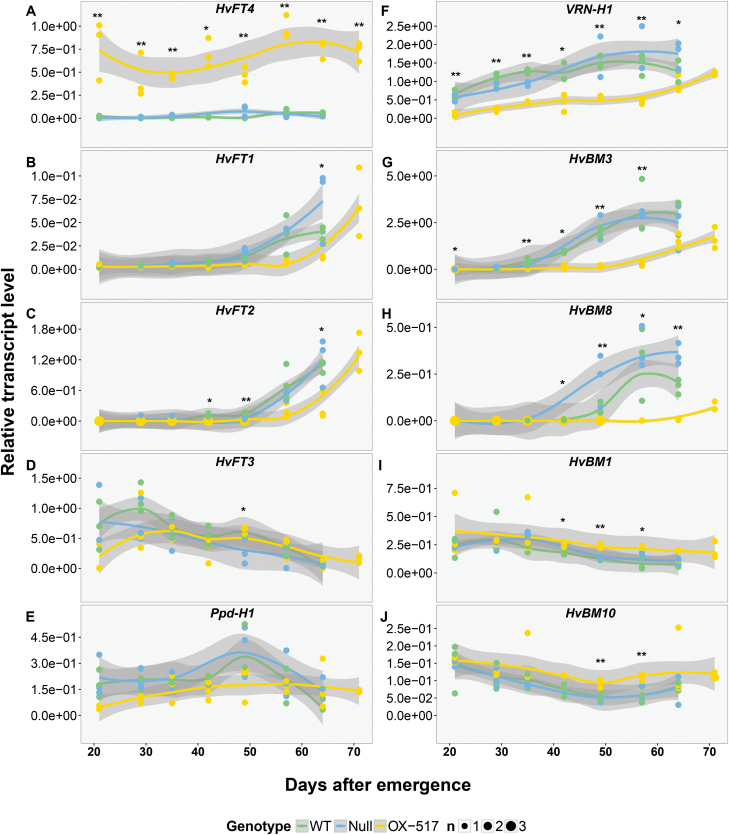
Temporal expression pattern of flowering time genes in the leaves of *Ubi::HvFT4*, the null segregant, and Golden Promise. Temporal expression of flowering time genes was assayed in the youngest fully emerged leaf of the main shoot of three plants of every genotype, every 7 d at Zeitgeber time T10 under long-day conditions (16 h light/8 h night). The expression level of each gene was normalized against the geometric mean of the reference genes *HvActin*, *HvGAPDH*, and *HvADP*. Polynomial regression models at a 95% confidence interval (Loess smooth line) are shown. For better visualization, asterisks instead of letters were used to indicate significant differences (*OX-517 differed significantly from one control line (WT or null), **OX-517 differed significantly from both control lines (WT and null). Corresponding letters are given in Supplementary Table S4. Statistical differences (*P*≤0.05) at each time point were calculated by one-way ANOVA followed by Tukey’s multiple comparison test (Tukey HSD). WT, Golden Promise; Null, null segregant; OX-517, *Ubi::HvFT4*-517.

To understand the role of HvFT4 in the control of flowering and identify genes which are regulated by HvFT4, the influence of *HvFT4* overexpression on the expression levels of known flowering time regulators was analyzed in the leaves and developing inflorescences (Fig. 4; Supplementary Table S4).

**Fig. 5.**
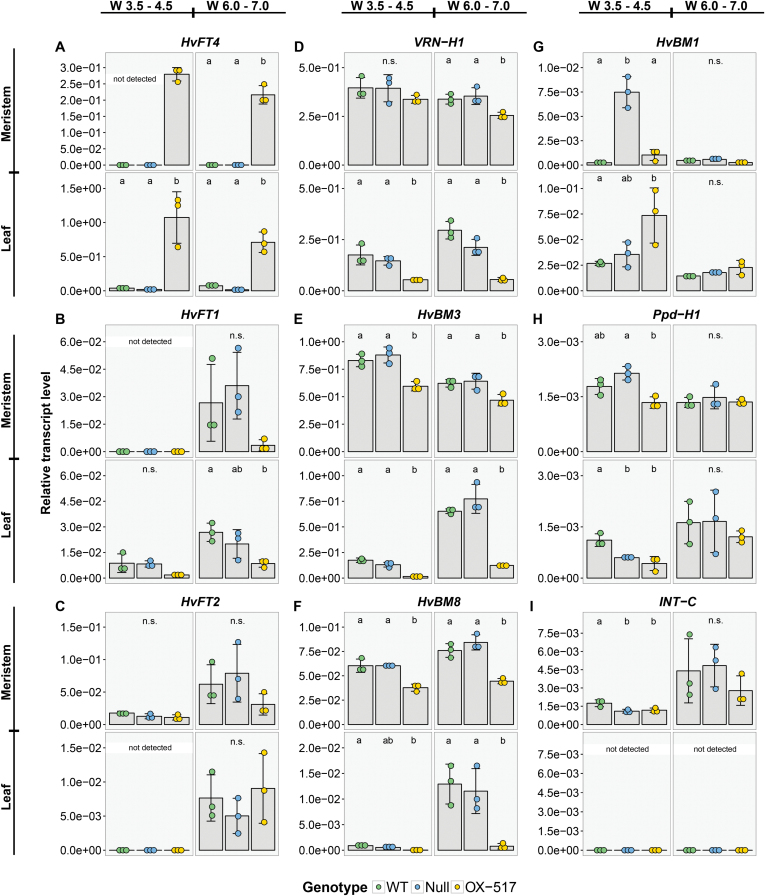
Influence of *Ubi::HvFT4* on the expression level of flowering-related genes in the leaves and meristem at Waddington stages 3.5–4.5 and 6.0–7.0. Expression of flowering time genes was assayed at two stages during reproductive development (W3.5–W4.5 and W6.0–W7.0) at Zeitgeber time T10 under long-day conditions (16 h light/8 h night). The expression level of each gene was normalized against the geometric mean of the reference genes *HvActin*, *HvGAPDH*, and *HvADP*. Each dot represents a pool of main shoot apices or leaf material from five single plants. Bars represent the mean ±SD. Statistical differences (*P*≤0.05) between genotypes were determined by one-way ANOVA followed by Tukey’s multiple comparison test (Tukey HSD). WT, Golden Promise; Null, null segregant; OX-517, *Ubi::HvFT4*-517.

Native *HvFT4* expression levels were very low in the leaf and strongly up-regulated in the transgenic plants (Figs 4A, 5A). *HvFT1* levels in the leaves were low in all plants and only started to increase after 49 DAE in the WT plants and after 60 DAE in the transgenic plants (Fig. 4B). Similarly, *HvFT2* expression levels in the leaves were below the detection limit until 42 DAE and 57 DAE when levels started increasing in the WT and transgenic lines, respectively (Fig. 4C). Expression levels of *HvFT1* and *HvFT2* were significantly different between *Ubi::HvFT4* and control plants at late reproductive stages (Fig. 4B, C). Overexpression of *HvFT4* had no significant effects on *HvFT3* and *Ppd-H1* expression levels in the leaves (Fig. 4D, E). We further analyzed the expression of the *AP1*-like genes *VRN-H1*, *HvBM3*, and *HvBM8* which are putative targets of *HvFT1* and *HvFT3* and promote floral development of barley (Hemming *et al.*, 2008; Digel *et al.*, 2015; Mulki *et al.*, 2018). *VRN-H1* was significantly down-regulated in the leaves of transgenic plants at all time points (Fig. 4F). *HvBM3* transcript levels were low, but detectable, in the leaves of all genotypes until 35 DAE and 50 DAE when expression levels increased in the WT and transgenic plants, respectively. *HvBM3* expression levels were significantly lower in transgenic plants compared with control plants at most time points (Fig. 4G). *HvBM8* expression levels in the leaves were below the detection levels until 42 DAE when they were strongly up-regulated in the WT, but not in transgenic plants, where *HvBM8* expression levels remained low until 60 DAE (Fig. 4H). In the inflorescence, *HvFT1* and *HvFT2* levels were below the detection limit at W3.5–4.5, but detectable at W6.0–7.0 when expression levels were higher in WT than in transgenic plants (Fig. 5B, C). Expression levels of *VRN-H1* were significantly down-regulated in the transgenic versus WT plants at W6.0–7.0, while *HvBM3* and *HvBM8* expression levels were significantly reduced in transgenic compared with WT plants at W3.5–4.0 and W6.0–7.0 (Fig. 5D–F). Additionally, the expression level of the barley MADS-box transcription factor and putative floral repressor genes *HvBM1* and *BM10* was measured (Hartmann *et al.*, 2000; Trevaskis *et al.*, 2007). While expression of *HvBM1* and *BM10* was significantly up-regulated in the leaf at late developmental stages, no significant differences were detected in the inflorescence (Figs 4I, J, 5G). *Ppd-H1* was expressed in the leaf and inflorescence, but no significant differences in expression levels were observed (Fig. 5H). As overexpression of *HvFT4* caused a strong reduction in tillering, we also assayed expression of *INTERMEDIUM-C* (*INT-C*), which is a repressor of axillary bud outgrowth (Ramsay *et al.*, 2011; Liller *et al.*, 2015). However, no effect of *HvFT4* overexpression on the expression of *INT-C* was observed either in the leaf or in the inflorescence (Fig. 5I).

Taken together, overexpression of *HvFT4* was associated with a down-regulation of *HvFT1* and *HvFT2* in the leaf, and of *VRN-H1*, *HvBM3*, and *HvBM8* in the leaf and inflorescence, and an up-regulation of *HvBM1* and *BM10* in the leaf.

To study natural variation at the *HvFT4* locus, we extracted and characterized SNP genotypes mapping to the *HvFT4* gene from the target enrichment data of 247 wild and 52 winter and spring domesticated barley accessions (Pankin *et al.*, 2018). The re-sequencing data covered 99% of the coding sequence of *HvFT4* on average and included intronic regions of the gene. The median joining network analysis based on 175 segregating SNPs identified 96 *HvFT4* haplotypes, among which 44 were found in more than a single barley accession (Supplementary Fig. S5). The *HvFT4* locus in domesticated barley was conserved compared with the wild genotypes and represented by four polymorphic haplotypes. All 23 winter barley and 19 spring cultivated barley genotypes carried the most frequent *HvFT4* haplotype 3. Haplotypes 1, 2, and 4 were found in the remaining 10 spring barley genotypes. Haplotypes 1 and 2 were shared between the wild and cultivated spring barley, indicating polyphyletic origins of the *HvFT4* locus in the domesticated genotypes descending from the wild barley populations from the North and South Levant areas of the Fertile Crescent. Based on the haplotype network, only two non-coding SNPs separated cultivated barley haplotypes 3 and 4 from the ancestral forms. Apparently, in the domesticated forms, no functional differentiation occurred in the coding sequences of the *HvFT4* alleles. Golden Promise (the background for the transformation) carried the *HvFT4* SNP haplotype 4 identical to Morex, which differed from the *HvFT4* haplotype 3 of Optic (a donor for the Ubi::*HvFT4* construct) by a single non-coding SNP as revealed by blast of the Morex *HvFT4* gene against Golden Promise pseudomolecules (Schreiber *et al.*, 2020). Within the wild barley genotypes, we detected seven non-synonymous substitutions. However, none of these affected the putatively critical amino acids, as described below and depicted in Supplementary Fig. S6.

### Multiple sequence alignment reveals amino acid substitutions in HvFT4 in conserved motifs

**Fig. 6.**
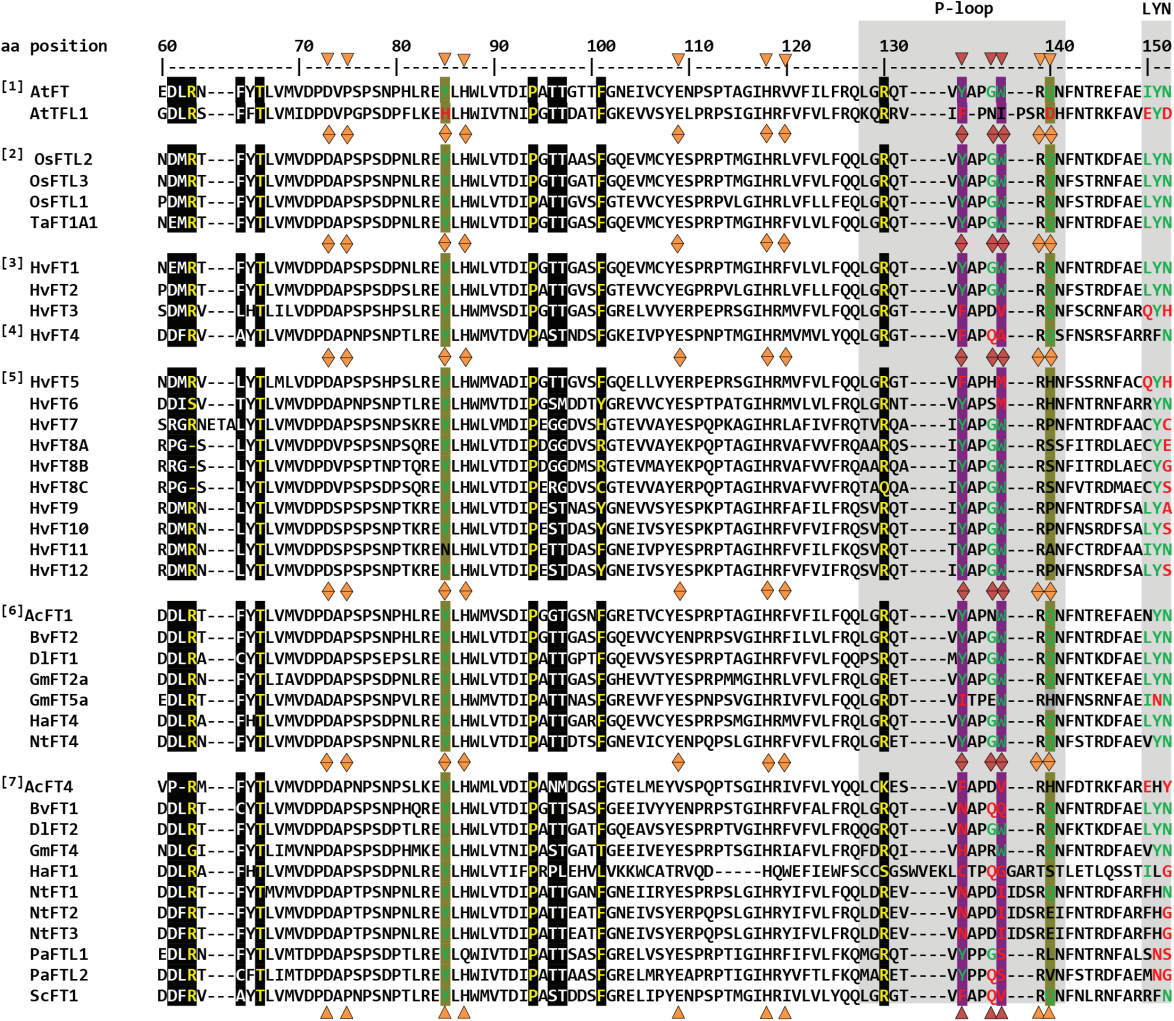
Inter- and intraspecies comparison of amino acid sequences of PEBP homologs. The protein sequences were grouped according to putative functions (inductive or repressive) and species groups (Arabidopsis, barley, other cereal crops, other species) to identify amino acid residues potentially linked to the inductive versus repressive function of PEBP-like genes across species. Group [1], inductive FT and TFL1 proteins from Arabidopsis; group [2], inductive rice and wheat FT-like proteins; group [3], inductive barley FT-like proteins; group [4], repressive barley FT-like proteins; group [5], uncharacterized barley FT-like proteins; group [6], inductive FT-like proteins from other species groups; [7], repressive FT-like proteins from other species. The P-loop (position 128–141) and LYN triad (position 150–153) of exon 4 are boxed in gray. The amino acid residues at position 85 and 140 which unambiguously distinguish between FT- and TFL1 homologs are highlighted in olive green (Hanzawa *et al.*, 2005; Ahn *et al.*, 2006). The amino acid residues at position 134 and 138, which are associated with inductive versus repressive function of FT-like proteins, are highlighted in purple (Wickland and Hanzawa, 2015). The amino acid residues at position 134, 138, and 137, which are responsible for the opposing functions of BvFT2 and BvFT1 in beet, are marked with a red triangle (Pin *et al.*, 2010). Amino acid residues which are specifically attributed to *FT*-like genes and/or are associated with flowering promotion are displayed in green letters. Amino acid residues which are specifically attributed to *TFL1*-like genes and/or are associated with flowering repression are displayed in red letters (Pin *et al.*, 2010; Klintenäs *et al.*, 2012; Wickland and Hanzawa, 2015). Amino acid residues with no characterized effect on flowering time are depicted in black letters. Amino acid residues lining the ligand-binding site as shown by Ahn *et al.* (2006) are marked with an orange triangle. Amino acid residues located at the binding interface with 14-3-3 protein are highlighted in black with white characters. Critical amino acid residues which abolished protein interactions between 14-3-3 proteins and TaFT1 or OsFTL2, respectively, in yeast two-hybrid assays when mutated are shown in yellow letters (Taoka *et al.*, 2011; Li *et al.*, 2015). All positions given refer to the amino acid position in Arabidopsis FT protein. Complete protein sequences were aligned with ClustalW. For better visualization, only the part of the PEBP domain is shown which includes amino acid residues critical for PEBP function.

The phenotypic and expression analysis suggested that HvFT4 acts as a repressor of flowering through down-regulation of floral promotors in the leaf and inflorescence. It is well known that small changes in individual amino acid residues encodesd by *FT*-like genes determine whether they act as repressors or activators of flowering (Ahn *et al.*, 2006; Kaneko-Suzuki *et al.*, 2018). We aimed to identify amino acid residues which might explain the repressive function of HvFT4 by aligning the HvFT4 protein sequence with protein sequences of *FT*-like genes from various species (Fig. 6; Supplementary Table S3).

Sequence comparison revealed that the amino acid residues R63, T67, P94, F101, and R130, previously determined to be critical for FT–14-3-3 interaction in wheat and rice (Taoka *et al.*, 2011; Li *et al.*, 2015), are conserved among barley HvFT1–HvFT4 (Fig. 6). In Arabidopsis, the signatures Y85 and Q140 differentiate the floral promotor FT from its sister protein TFL1 which acts as a repressor of flowering (Hanzawa *et al.*, 2005; Ahn *et al.*, 2006). However, HvFT4 shares these functionally important FT signatures with FT and floral repressors from other species, but not the repressor TFL1 (Fig. 6). The LYN triad, which is immediately adjacent to and makes contact with the segment B external loop in Arabidopsis (Ahn *et al.*, 2006), is conserved in HvFT1, HvFT2, and other FT-like proteins which function as floral promoters (Fig. 6). It is, however, more variable in proteins characterized as floral repressors including HvFT4 (Fig. 6). Remarkably, HvFT4 shows the same amino acid pattern RFN at the LYN triad as ScFT1, another putative FT-like floral repressor from the Poaceae species sugarcane (*Saccharum* spp.) (Coelho *et al.*, 2014).

All the candidate amino acid residues putatively critical for the HvFT4 function (R63, T67, P94, F101, R130, and Y85) were conserved across 32 FT4 protein sequences from 24 grass monocot species (Supplementary Fig. S6; Supplementary Table S2). While wheat contains two close homologs designated as TaFT4-A1 and TaFT4-A2 (Halliwell *et al.* 2016), only one FT4 gene was identified in barley (Supplementary Fig. S6). The FT4 orthologs were not present in the genomes of monocot species outside of the grass clade such as pineapple (*Ananas comosus*), banana (*Musa acuminata*), and white yam (*Dioscorea rotundata*).

Taken together, the sequence comparison suggested candidate amino acid residues that might confer the repressive activity of HvFT4. Which of these residues is causative still needs to be elucidated.

## Discussion

In this study, *HvFT4* was overexpressed in the genetic background of the spring cultivar Golden Promise under LDs to investigate the influence of HvFT4 on flowering time and inflorescence development in barley. Overexpression of *HvFT4* prolonged vegetative growth and delayed spikelet initiation (Figs 1, 2). The effects of HvFT4 were antagonistic to those of HvFT1, HvFT2, and HvFT3, which promote spikelet initiation and inflorescence development in barley (Digel *et al.*, 2015, Mulki *et al.*, 2018; Shaw *et al.*, 2019). Typically, a delay in reproductive development and reduction in apical dominance are associated with an increase in the number of spikelets and tillers in barley (Digel *et al.*, 2015; Bi *et al.*, 2019). However, overexpression of *HvFT4* delayed reproductive development, decreased the number of spikelets on the main culm, and reduced the number of tillers and grain weight (Fig. 3; Supplementary Figs S1, S2). Consequently, overexpression of *HvFT4* had negative pleiotropic effects on a number of reproductive traits in barley. These effects of HvFT4 on apical, axillary, and spikelet meristems suggested that HvFT4 plays a role in the development of different shoot meristems. Tsuji *et al.* (2015) already showed that *Hd3a*, an *HvFT1* ortholog in rice, promotes the development not only of the apical meristem, but also that of lateral buds and therefore influences branching in rice. Similarly, mutations in *CENTRORADIALIS* (*HvCEN*), a barley homolog of the floral repressor *TFL1*, reduced tiller number, and spikelet and grain number per spike (Bi *et al.*, 2019). Furthermore, a key regulator of axillary bud outgrowth is *TEOSINTE BRANCHED 1* (*TB1*). Homologs of the maize (*Zea mays*) *TB1* gene, *INTERMEDIUM-C* (*INT-C*) in barley and *BRANCHED 1* (*BRC1*) in Arabidopsis, suppress axillary bud outgrowth (Doebley *et al.*, 1997; Hubbard *et al.*, 2002). In Arabidopsis, BRC1 was shown to interact with the FT protein to delay floral transition in the axillary meristems (Niwa *et al.*, 2013). Similarly, it was recently shown that in wheat the TB1 protein interacts with FT1 and that increased dosage of TB1 alters inflorescence architecture and tiller number (Dixon *et al.*, 2018). While overexpression of *HvFT4* did not affect *INT-C* expression (Fig. 5), HvFT4 protein might interact with INT-C and thereby affect the TB1/FT1 dosage and thus tiller outgrowth and inflorescence architecture. Similarly, it was shown in Arabidopsis that overexpression of *BROTHER OF FT AND TFL1* (*BFT*), whose product shares a higher sequence similarity with FT than with TFL1, resulted in late flowering and suppression of axillary inflorescence growth (Kobayashi *et al.*, 1999; Yoo *et al.*, 2010). However, the results obtained by the constitutive overexpression of *HvFT4* need to be interpreted with caution. It is also possible that the reduced number of tillers observed in *Ubi::HvFT4* plants resulted from ectopic expression of *HvFT4* in tissue where it is usually not expressed. For example, we only observed expression of the native *HvFT4* in the leaves, but not in the inflorescence (Fig. 5).

Overexpression of *HvFT4* was associated with a strong down-regulation of the *AP1-*like genes *VRN-H1*, *HvBM3*, and *HvBM8* in the leaves and meristem (Fig. 4; Fig. 5). In rice, simultaneous knockdown of *OsMADS14* (*VRN1*, *FUL1*), *OsMADS15* (*BM3*, *FUL2*), and *OsMAD18* (*BM8*, *FUL3*) impaired spikelet development and resulted in floral reversion (Kobayashi *et al.*, 2012). Similarly, triple wheat *vrn1ful2ful3* mutants developed vegetative tillers instead of spikelets (Li *et al.*, 2019). Low expression levels of *VRN-H1*, *BM3*, and *BM8* in *Ubi::HvFT4* plants might have caused the delay in spikelet initiation, and reduced floret fertility and grain set. It has already been demonstrated that *FT1*, *FT2*, and the *BM* genes affect floret fertility and grain set (Digel *et al.*, 2015; Ejaz *et al.*, 2017; Shaw *et al.*, 2019). We therefore conclude that the down-regulation of *BM*-like genes and consequently *HvFT1* and *HvFT2* in the inflorescence has contributed to the reduction in fertility and grain set of the main and axillary shoots.

Evidence from Arabidopsis shows that FT and TFL1 act as antagonists by competing for the same binding partners (Ahn *et al.*, 2006). It was proposed that FT recruits a transcriptional activator, whereas TFL1 recruits a transcriptional repressor (Hanzawa *et al.*, 2005; Ahn *et al.*, 2006; Hanano and Goto, 2011; Ho and Weigel, 2014). HvFT4 might also compete with HvFT1, HvFT2, and HvFT3 for binding partners and thus control the expression of putative target genes such as the *BM* genes. Studies in maize and *Brachypodium* further support this hypothesis. Maize plants with reduced expression of the floral promoter *FT*-like gene *Zea CENTRORADIALIS 8* (*ZCN8*) resembled the phenotype of plants which overexpressed the floral repressor *TFL1*-like *Zea CENTRORADIALIS 2* (*ZCN2*), suggesting that both proteins compete for the same interaction partners (Danilevskaya *et al.*, 2011). In *Brachypodium*, an SD-induced FLOWERING LOCUS T ortholog, FT-like 9 (FTL9), promotes flowering in SDs but inhibits flowering in LDs (Qin *et al.*, 2019). Both proteins could interact with FD1 to form a flowering activation complex (FAC) but with lower activity of FTL9-FAC than of FT1-FAC. This probably resulted in a positive role for FTL9 in promoting floral transition under SDs when FT1 is not expressed, but a dominant-negative role when FT1 accumulates under LDs. Consequently, we propose that HvFT4 may also compete with HvFT-like proteins that act as floral activators to repress flowering under LDs. However, further experimental evidence is needed to analyse the activity and binding partners of the HvFT4 protein (Li *et al.*, 2015).

Sequence comparison of FT-like proteins suggested several residues that might be causal for the repressive function of HvFT4 (Fig. 6). The amino acid residues critical for FT–14-3-3 interaction were conserved in barley HvFT4, suggesting that HvFT4 possesses 14-3-3 binding capacities and engages in a normal FAC formation. Indeed, HvFT1, HvFT3, and HvFT4 were shown to interact with the same 14-3-3 proteins in a yeast two-hybrid assay (Li *et al.*, 2015). Secondly, HvFT4 and HvFT3 proteins carry amino acid substitutions within their P-loop, among others, at the amino acid positions 134, 137, and 138. Interestingly, these three amino acids were identified to be the major cause of antagonistic functions of two FT-like proteins in beet (Pin *et al.*, 2010). The exchange of the external loop of BvFT1 for the P-loop of BvFT2, with the three altered amino acids, converted BvFT1 from a floral repressor to an activator (Pin *et al.*, 2010). Remarkably, HvFT4, but not HvFT3, shares a glutamine at position 137 with the flowering repressor BvFT1. However, additional experiments with single amino acid swapping indicated that Y134, W138, and Y85 are essential for the inductive function of *FT*-like genes in beet (Klintenäs *et al.*, 2012).

Furthermore, HvFT3 and HvFT4 do not carry the conserved amino acids Y134 and W138, which are associated with FT-like flowering inductive function, but rather hydrophobic amino acids which they share with the flowering repressor TFL1 (Wickland and Hanzawa, 2015). It is still not known which amino acid position is critical for flowering promotive or repressive function in barley and which substitutions are tolerated or lead to modification of the protein function. However, HvFT4 sequence features correspond to those of other known repressor *FT* genes and are indicative of candidate amino acid residues that might confer the repressive activity of HvFT4. The amino acid residues responsible for the antagonistic function in the closely related *FT*-like genes should be tested using base editing, a novel genome editing approach that directly converts one base or base pair into another, enabling the efficient installation of point mutations in non-dividing cells without generating excess undesired editing byproducts (Rees and Liu 2018).

Taken together, our study provided phenotypic and molecular data that indicate that *HvFT4* may act as a repressor of reproductive development in barley. Plants overexpressing *HvFT4* displayed a delay in reproductive development, a reduction in spikelet and tiller number and floret fertility, and a down-regulation of genes promoting spikelet and floral development.

## Supplementary data

The following supplementary data are available at *JXB* online.

Table S1. List of primers used in this study.

Table S2. FT4 orthologs in the genomes of 24 monocot species.

Table S3. Protein IDs or gene model information used for the multiple sequence alignment.

Table S4. Significant differences for temporal and developmental expression levels of flowering time genes in the leaves of *Ubi::HvFT4*, null segregant, and Golden Promise plants (Fig. 4).

Fig. S1. Overexpression of *HvFT4* decreases tillering and increases the number of leaves on the main shoot, but does not influence plant height and peduncle extrusion.

Fig. S2. Overexpression of *HvFT4* reduces floret fertility of the main shoot spike.

Fig. S3. Overexpression of *HvFT4* does not influence leaf size.

Fig. S4. Overexpression of *HvFT4* reduces grain weight and width.

Fig. S5. A median joining haplotype network of 96 *HvFT4* haplotypes.

Fig. S6. Multiple alignment of the FT4 orthologs from 24 monocot grass species.

eraa466_suppl_Supplementary_File001

## Data Availability

All data supporting the findings of this study are available within the paper and within its supplementary data published online.

## References

[CIT0001] Abe M, Kobayashi Y, Yamamoto S, Daimon Y, Yamaguchi A, Ikeda Y, Ichinoki H, Notaguchi M, Goto K, Araki T 2005 FD, a bZIP protein mediating signals from the floral pathway integrator FT at the shoot apex. Science 309, 1052–1056. 16099979 10.1126/science.1115983

[CIT0002] Ahn JH, Miller D, Winter VJ, Banfield MJ, Lee JH, Yoo SY, Henz SR, Brady RL, Weigel D 2006 A divergent external loop confers antagonistic activity on floral regulators FT and TFL1. The EMBO Journal 25, 605–614. 16424903 10.1038/sj.emboj.7600950 PMC1383534

[CIT0003] Altschul SF, Gish W, Miller W, Myers EW, Lipman DJ 1990 Basic local alignment search tool. Journal of Molecular Biology 215, 403–410. 2231712 10.1016/S0022-2836(05)80360-2

[CIT0004] Andrés F, Coupland G 2012 The genetic basis of flowering responses to seasonal cues. Nature Reviews. Genetics 13, 627–639. 10.1038/nrg3291 22898651

[CIT0005] Bandelt HJ, Forster P, Röhl A 1999 Median-joining networks for inferring intraspecific phylogenies. Molecular Biology and Evolution 16, 37–48. 10331250 10.1093/oxfordjournals.molbev.a026036

[CIT0006] Bi X, van Esse W, Mulki MA, Kirschner G, Zhong J, Simon R, von Korff M 2019 CENTRORADIALIS interacts with FLOWERING LOCUS T-like genes to control floret development and grain number. Plant Physiology 180, 1013–1030. 31004004 10.1104/pp.18.01454 PMC6548242

[CIT0007] Bolser DM, Staines DM, Perry E, Kersey PJ 2017 Ensembl plants: integrating tools for visualizing, mining, and analyzing plant genomic data. Methods in Molecular Biology 1533, 1–31. 27987162 10.1007/978-1-4939-6658-5_1

[CIT0008] Bradbury PJ, Zhang Z, Kroon DE, Casstevens TM, Ramdoss Y, Buckler ES 2007 TASSEL: software for association mapping of complex traits in diverse samples. Bioinformatics 23, 2633–2635. 17586829 10.1093/bioinformatics/btm308

[CIT0009] Campoli C, Drosse B, Searle I, Coupland G, von Korff M 2012 Functional characterisation of HvCO1, the barley (*Hordeum vulgare*) flowering time ortholog of CONSTANS: functional characterisation of HvCO1 in barley. The Plant Journal 69, 868–880. 22040323 10.1111/j.1365-313X.2011.04839.x

[CIT0010] Campoli C, von Korff M 2014 Genetic control of reproductive development in temperate cereals. Advances in Botanical Research 72, 131–158.

[CIT0011] Chardon F, Damerval C 2005 Phylogenomic analysis of the PEBP gene family in cereals. Journal of Molecular Evolution 61, 579–590 16170456 10.1007/s00239-004-0179-4

[CIT0012] Cockram J, Jones H, Leigh FJ, O’Sullivan D, Powell W, Laurie DA, Greenland AJ 2007 Control of flowering time in temperate cereals: genes, domestication, and sustainable productivity. Journal of Experimental Botany 58, 1231–1244. 17420173 10.1093/jxb/erm042

[CIT0013] Coelho CP, Minow MAA, Chalfun-Júnior A, Colasanti J 2014 Putative sugarcane FT/TFL1 genes delay flowering time and alter reproductive architecture in Arabidopsis. Frontiers of Plant Sciences 5, 221. 10.3389/fpls.2014.00221 PMC4033272 24904616

[CIT0014] Comadran J, Kilian B, Russell J, et al. 2012 Natural variation in a homolog of Antirrhinum CENTRORADIALIS contributed to spring growth habit and environmental adaptation in cultivated barley. Nature Genetics 44, 1388. 23160098 10.1038/ng.2447

[CIT0015] Corbesier L, Vincent C, Jang S, et al. 2007 FT protein movement contributes to long-distance signaling in floral induction of arabidopsis. Science 316, 1030–1033. 17446353 10.1126/science.1141752

[CIT0016] Danilevskaya ON, Meng X, Hou Z, Ananiev EV, Simmons CR 2008 A genomic and expression compendium of the expanded PEBP gene family from maize. Plant Physiology 146, 250–264. 17993543 10.1104/pp.107.109538 PMC2230559

[CIT0017] Danilevskaya ON, Meng X, McGonigle B, Muszynski MG 2011 Beyond flowering time. Plant Signaling & Behavior 6, 1267–1270. 21847027 10.4161/psb.6.9.16423 PMC3258048

[CIT0018] Digel B, Pankin A, von Korff M 2015 Global transcriptome profiling of developing leaf and shoot apices reveals distinct genetic and environmental control of floral transition and inflorescence development in barley. The Plant Cell 27, 2318–2334. 26307377 10.1105/tpc.15.00203 PMC4815099

[CIT0019] Dixon LE, Greenwood JR, Bencivenga S, Zhang P, Cockram J, Mellers G, Ramm K, Cavanagh C, Swain SM, Boden SA 2018 TEOSINTE BRANCHED1 regulates inflorescence architecture and development in bread wheat (*Triticum aestivum*). The Plant Cell 30, 563–581. 29444813 10.1105/tpc.17.00961 PMC5894836

[CIT0020] Doebley J, Stec A, Hubbard L 1997 The evolution of apical dominance in maize. Nature 386, 485. 9087405 10.1038/386485a0

[CIT0021] Ejaz M, von Korff M 2017 The genetic control of reproductive development under high ambient temperature. Plant Physiology 173, 294–306. 28049855 10.1104/pp.16.01275 PMC5210726

[CIT0022] Faure S, Higgins J, Turner A, Laurie DA 2007 The FLOWERING LOCUS T-like gene family in barley (*Hordeum vulgare*). Genetics 176, 599–609. 17339225 10.1534/genetics.106.069500 PMC1893030

[CIT0023] Fornara F, de Montaigu A, Coupland G 2010 SnapShot: control of flowering in Arabidopsis. Cell 141, 550. 20434991 10.1016/j.cell.2010.04.024

[CIT0024] Gol L, Haraldsson EB, von Korff M 2021 *Ppd-H1* integrates drought stress signals to control spike development and flowering time in barley. Journal of Experimental Botany 72, 122–136. 32459309 10.1093/jxb/eraa261 PMC7816852

[CIT0025] Gol L, Tomé F, von Korff M 2017 Floral transitions in wheat and barley: interactions between photoperiod, abiotic stresses, and nutrient status. Journal of Experimental Botany 68, 1399–1410. 28431134 10.1093/jxb/erx055

[CIT0026] Halliwell J, Borrill P, Gordon A, Kowalczyk R, Pagano ML, Saccomanno B, Bentley AR, Uauy C, Cockram J 2016 Systematic investigation of FLOWERING LOCUS T-like poaceae gene families identifies the short-day expressed flowering pathway gene, TaFT3 in wheat (*Triticum aestivum* L.). Frontiers in Plant Science 7, 857. 27458461 10.3389/fpls.2016.00857 PMC4937749

[CIT0027] Hanano S, Goto K 2011 Arabidopsis TERMINAL FLOWER1 is involved in the regulation of flowering time and inflorescence development through transcriptional repression. The Plant Cell 23, 3172–3184. 21890645 10.1105/tpc.111.088641 PMC3203435

[CIT0028] Hanzawa Y, Money T, Bradley D 2005 A single amino acid converts a repressor to an activator of flowering. Proceedings of the National Academy of Sciences, USA 102, 7748–7753. 10.1073/pnas.0500932102 PMC1140427 15894619

[CIT0029] Hartmann U, Höhmann S, Nettesheim K, Wisman E, Saedler H, Huijser P 2000 Molecular cloning of SVP: a negative regulator of the floral transition in Arabidopsis. The Plant Journal 21, 351–360. 10758486 10.1046/j.1365-313x.2000.00682.x

[CIT0030] Hayama R, Yokoi S, Tamaki S, Yano M, Shimamoto K 2003 Adaptation of photoperiodic control pathways produces short-day flowering in rice. Nature 422, 719–722. 12700762 10.1038/nature01549

[CIT0031] Hemming MN, Fieg S, Peacock WJ, Dennis ES, Trevaskis B 2009 Regions associated with repression of the barley (*Hordeum vulgare*) VERNALIZATION1 gene are not required for cold induction. Molecular Genetics and Genomics 282, 107–117. 19404679 10.1007/s00438-009-0449-3

[CIT0032] Hemming MN, Peacock WJ, Dennis ES, Trevaskis B 2008 Low-temperature and daylength cues are integrated to regulate FLOWERING LOCUS T in barley. Plant Physiology 147, 355–366. 18359843 10.1104/pp.108.116418 PMC2330320

[CIT0033] Ho WW, Weigel D 2014 Structural features determining flower-promoting activity of Arabidopsis FLOWERING LOCUS T. The Plant Cell 26, 552–564. 24532592 10.1105/tpc.113.115220 PMC3967025

[CIT0034] Hsu CY, Adams JP, Kim H, et al. 2011 FLOWERING LOCUS T duplication coordinates reproductive and vegetative growth in perennial poplar. Proceedings of the National Academy of Sciences, USA 108, 10756–10761. 10.1073/pnas.1104713108 PMC3127867 21653885

[CIT0035] Hubbard L, McSteen P, Doebley J, Hake S 2002 Expression patterns and mutant phenotype of teosinte branched1 correlate with growth suppression in maize and teosinte. Genetics 162, 1927–1935. 12524360 10.1093/genetics/162.4.1927 PMC1462370

[CIT0036] Kaneko-Suzuki M, Kurihara-Ishikawa R, Okushita-Terakawa C, Kojima C, Nagano-Fujiwara M, Ohki I, Tsuji H, Shimamoto K, Taoka KI 2018 TFL1-like proteins in rice antagonize rice FT-like protein in inflorescence development by competition for complex formation with 14-3-3 and FD. Plant & Cell Physiology 59, 458–468. 29401229 10.1093/pcp/pcy021

[CIT0037] Kardailsky I, Shukla VK, Ahn JH, Dagenais N, Christensen SK, Nguyen JT, Chory J, Harrison MJ, Weigel D 1999 Activation tagging of the floral inducer FT. Science 286, 1962–1965. 10583961 10.1126/science.286.5446.1962

[CIT0038] Kikuchi R, Kawahigashi H, Ando T, Tonooka T, Handa H 2009 Molecular and functional characterization of PEBP genes in barley reveal the diversification of their roles in flowering. Plant Physiology 149, 1341–1353. 19168644 10.1104/pp.108.132134 PMC2649388

[CIT0039] Klintenäs M, Pin PA, Benlloch R, Ingvarsson PK, Nilsson O 2012 Analysis of conifer FLOWERING LOCUS T/TERMINAL FLOWER1-like genes provides evidence for dramatic biochemical evolution in the angiosperm FT lineage. New Phytologist 196, 1260–1273. 10.1111/j.1469-8137.2012.04332.x 23020222

[CIT0040] Kobayashi Y, Kaya H, Goto K, Iwabuchi M, Araki T 1999 A pair of related genes with antagonistic roles in mediating flowering signals. Science 286, 1960–1962. 10583960 10.1126/science.286.5446.1960

[CIT0041] Kobayashi K, Yasuno N, Sato Y, Yoda M, Yamazaki R, Kimizu M, Yoshida H, Nagamura Y, Kyozuka J 2012 Inflorescence meristem identity in rice is specified by overlapping functions of three AP1/FUL-like MADS box genes and PAP2, a SEPALLATA MADS box gene. The Plant Cell 24, 1848–1859. 22570445 10.1105/tpc.112.097105 PMC3442573

[CIT0042] Kojima S, Takahashi Y, Kobayashi Y, Monna L, Sasaki T, Araki T, Yano M 2002 *Hd3a*, a rice ortholog of the Arabidopsis *FT* gene, promotes transition to flowering downstream of Hd1 under short-day conditions. Plant & Cell Physiology 43, 1096–1105. 12407188 10.1093/pcp/pcf156

[CIT0043] Komiya R, Ikegami A, Tamaki S, Yokoi S, Shimamoto K 2008 Hd3a and RFT1 are essential for flowering in rice. Development 135, 767–774. 18223202 10.1242/dev.008631

[CIT0044] Larsson A 2014 AliView: a fast and lightweight alignment viewer and editor for large datasets. Bioinformatics 30, 3276–3278. 25095880 10.1093/bioinformatics/btu531 PMC4221126

[CIT0045] Laurie DA, Pratchett N, Snape JW, Bezant JH 1995 RFLP mapping of five major genes and eight quantitative trait loci controlling flowering time in a winter×spring barley (*Hordeum vulgare* L.) cross. Genome 38, 575–585. 18470191 10.1139/g95-074

[CIT0046] Leigh JW, Bryant D 2015 popart: full-feature software for haplotype network construction. Methods in Ecology and Evolution 6, 1110–1116.

[CIT0047] Li C, Lin H, Chen A, Lau M, Jernstedt J, Dubcovsky J 2019 Wheat VRN1, FUL2 and FUL3 play critical and redundant roles in spikelet development and spike determinacy. Development 146, dev175398. 10.1242/dev.175398 PMC6679363 31337701

[CIT0048] Li C, Lin H, Dubcovsky J 2015 Factorial combinations of protein interactions generate a multiplicity of florigen activation complexes in wheat and barley. The Plant Journal 84, 70–82. 26252567 10.1111/tpj.12960 PMC5104200

[CIT0049] Liller CB, Neuhaus R, von Korff M, Koornneef M, van Esse W 2015 Mutations in barley row type genes have pleiotropic effects on shoot branching. PLoS One 10, e0140246. 26465604 10.1371/journal.pone.0140246 PMC4605766

[CIT0050] Lv B, Nitcher R, Han X, Wang S, Ni F, Li K, Pearce S, Wu J, Dubcovsky J, Fu D 2014 Characterization of FLOWERING LOCUS T1 (FT1) gene in Brachypodium and wheat. PLoS One 9, e94171. 24718312 10.1371/journal.pone.0094171 PMC3981775

[CIT0051] Money D, Gardner K, Migicovsky Z, Schwaninger H, Zhong GY, Myles S 2015 LinkImpute: fast and accurate genotype imputation for nonmodel organisms. G3 (Bethesda, Md.) 5, 2383–2390. 10.1534/g3.115.021667 PMC4632058 26377960

[CIT0052] Mouradov A, Cremer F, Coupland G 2002 Control of flowering time: interacting pathways as a basis for diversity. The Plant Cell 14, S111–S130. 12045273 10.1105/tpc.001362 PMC151251

[CIT0053] Mulki MA, Bi X, von Korff M 2018 FLOWERING LOCUS T3 controls spikelet initiation but not floral development. Plant Physiology 178, 1170–1186. 30213796 10.1104/pp.18.00236 PMC6236595

[CIT0054] Mulki MA, von Korff M 2016 CONSTANS controls floral repression by up-regulating VERNALIZATION2 (VRN-H2) in barley. Plant Physiology 170, 325–337. 26556793 10.1104/pp.15.01350 PMC4704585

[CIT0055] Niwa M, Daimon Y, Kurotani K, et al. 2013 BRANCHED1 interacts with FLOWERING LOCUS T to repress the floral transition of the axillary meristems in Arabidopsis. The Plant Cell 25, 1228–1242. 23613197 10.1105/tpc.112.109090 PMC3663264

[CIT0056] Pankin A, Altmüller J, Becker C, von Korff M 2018 Targeted resequencing reveals genomic signatures of barley domestication. New Phytologist 218, 1247–1259. 10.1111/nph.15077 PMC5947139 29528492

[CIT0057] Pin PA, Benlloch R, Bonnet D, Wremerth-Weich E, Kraft T, Gielen JJ, Nilsson O 2010 An antagonistic pair of FT homologs mediates the control of flowering time in sugar beet. Science 330, 1397–1400. 21127254 10.1126/science.1197004

[CIT0058] Qin Z, Bai Y, Muhammad S, Wu X, Deng P, Wu J, An H, Wu L 2019 Divergent roles of FT-like 9 in flowering transition under different day lengths in *Brachypodium distachyon*. Nature Communications 10, 812. 10.1038/s41467-019-08785-y PMC6379408 30778068

[CIT0059] R Development Core Team. 2008 R: a language and environment for statistical computing. Vienna: R Foundation for Statistical Computing.

[CIT0060] Ramsay L, Comadran J, Druka A, et al. 2011 INTERMEDIUM-C, a modifier of lateral spikelet fertility in barley, is an ortholog of the maize domestication gene TEOSINTE BRANCHED 1. Nature Genetics 43, 169–172. 21217754 10.1038/ng.745

[CIT0061] Rees HA, Liu DR 2018 Base editing: precision chemistry on the genome and transcriptome of living cells. Nature Reviews Genetics 19, 770–788. 10.1038/s41576-018-0059-1 PMC6535181 30323312

[CIT0062] Rollins JA, Drosse B, Mulki MA, Grando S, Baum M, Singh M, Ceccarelli S, von Korff M 2013 Variation at the vernalisation genes Vrn H1 and Vrn H2 determines growth and yield stability in barley (*Hordeum vulgare*) grown under dryland conditions in Syria. Theoretical and Applied Genetics 126, 2803–2824 23918065 10.1007/s00122-013-2173-y

[CIT0063] Schreiber M, Mascher M, Wright J, Padmarasu S, Himmelbach A, Heavens D, Milne L, Clavijo BJ, Stein N, Waugh R 2020 A genome assembly of the barley ‘Transformation Reference’ cultivar golden promise. G3 (Bethesda, Md.) 10, 1823–1827. 10.1534/g3.119.401010 PMC7263683 32241919

[CIT0064] Shaw LM, Lyu B, Turner R, Li C, Chen F, Han X, Fu D, Dubcovsky J 2019 FLOWERING LOCUS T2 (FT2) regulates spike development and fertility in temperate cereals. Journal of Experimental Botany 70, 193–204 30295847 10.1093/jxb/ery350 PMC6305198

[CIT0065] Tamaki S, Matsuo S, Wong HL, Yokoi S, Shimamoto K 2007 Hd3a protein is a mobile flowering signal in rice. Science 316, 1033–1036. 17446351 10.1126/science.1141753

[CIT0066] Taoka K, Ohki I, Tsuji H, et al. 2011 14-3-3 proteins act as intracellular receptors for rice Hd3a florigen. Nature 476, 332–335. 21804566 10.1038/nature10272

[CIT0067] Trevaskis B, Tadege M, Hemming MN, Peacock WJ, Dennis ES, Sheldon C 2007 Short vegetative phase-like MADS-box genes inhibit floral meristem identity in barley. Plant Physiology 143, 225–235. 17114273 10.1104/pp.106.090860 PMC1761976

[CIT0068] Tsuji H, Tachibana C, Tamaki S, Taoka K, Kyozuka J, Shimamoto K 2015 Hd3a promotes lateral branching in rice. The Plant Journal 82, 256–266. 25740115 10.1111/tpj.12811

[CIT0069] Turner A, Beales J, Faure S, Dunford RP, Laurie DA 2005 The pseudo-response regulator Ppd-H1 provides adaptation to photoperiod in barley species. Science 310, 1029–1031. 16284181 10.1126/science.1117619

[CIT0070] Verhoeven KJ, Poorter H, Nevo E, Biere A 2008 Habitat-specific natural selection at a flowering-time QTL is a main driver of local adaptation in two wild barley populations. Molecular Ecology 17, 3416–3424. 18573164 10.1111/j.1365-294X.2008.03847.x

[CIT0071] Waddington SR, Cartwright PM, Wall PC 1983 A quantitative scale of spike initial and pistil development in barley and wheat. Annals of Botany 51, 119–130.

[CIT0072] Wickland DP, Hanzawa Y 2015 The FLOWERING LOCUS T/TERMINAL FLOWER 1 gene family: functional evolution and molecular mechanisms. Molecular Plant 8, 983–997. 25598141 10.1016/j.molp.2015.01.007

[CIT0073] Wigge PA, Kim MC, Jaeger KE, Busch W, Schmid M, Lohmann JU, Weigel D 2005 Integration of spatial and temporal information during floral induction in Arabidopsis. Science 309, 1056–1059. 16099980 10.1126/science.1114358

[CIT0074] Yan L, Fu D, Li C, Blechl A, Tranquilli G, Bonafede M, Sanchez A, Valarik M, Yasuda S, Dubcovsky J 2006 The wheat and barley vernalization gene VRN3 is an orthologue of FT. Proceedings of the National Academy of Sciences, USA 103, 19581–19586. 10.1073/pnas.0607142103 PMC1748268 17158798

[CIT0075] Yan L, Loukoianov A, Blechl A, Tranquilli G, Ramakrishna W, SanMiguel P, Bennetzen JL, Echenique V, Dubcovsky J 2004 The wheat VRN2 gene is a flowering repressor down-regulated by vernalization. Science 303, 1640–1644. 15016992 10.1126/science.1094305 PMC4737501

[CIT0076] Yan L, Loukoianov A, Tranquilli G, Helguera M, Fahima T, Dubcovsky J 2003 Positional cloning of the wheat vernalization gene VRN1. Proceedings of the National Academy of Sciences, USA 100, 6263–6268. 10.1073/pnas.0937399100 PMC156360 12730378

[CIT0077] Yoo SJ, Chung KS, Jung SH, Yoo SY, Lee JS, Ahn JH 2010 BROTHER OF FT AND TFL1 (BFT) has TFL1-like activity and functions redundantly with TFL1 in inflorescence meristem development in Arabidopsis. The Plant Journal 63, 241–253. 20409005 10.1111/j.1365-313X.2010.04234.x

[CIT0078] Zadoks JC, Chang TT, Konzak CF 1974 A decimal code for the growth stages of cereals. Weed Research 14, 415–421.

