# Supplementary material for: FLOWERING LOCUS T4 delays flowering and decreases floret fertility in barley
Source: J Exp Bot. 2020 Oct 13;72(1):107–21. doi: 10.1093/jxb/eraa466 (PMC7816854; doi:10.1093/jxb/eraa466)
Supplement: eraa466_suppl_Supplementary_File001 [file eraa466_suppl_supplementary_file001.pdf]

Supplementary Tables

Supplementary Table 1: List of primers used in this study.

Gene	Gene ID	Primer Sequences (Forward, Reverse)	Purpose	Reference
Ubi::HvFT4	***DQ411320.1***	CGT TGA GAT TGG TGG TGA TG	Genotyping	unpublished
		ATC TGC AGG TCG AAC GGT AT		
Hygromycin resistance		GCG CGC GAT AAT TTA GTC CTA GTT TGC G	Genotyping	Campoli et al. (2012a)
		ACG CGG ATT TCG GCT CCA ACA ATG		
VRN-H1	***AY750995***	AAT ACG ACT CAC TAT AGG GGC CGC TGC CCA A[illegible]	Geno-typing	von Zitzewitz et al. (2005)
		ATC CCC TCC TCC CAT GTT CG		
HvActin	***AY145451***	CGT GTT GGA TTC TGG TGA TG	qRT-PCR	Campoli et al. (2012a)
		AGC CAC ATA TGC GAG CTT CT		
HvGAPDH	***AK362208***	GTG AGG CTG GTG CTG ATT ACG	qRT-PCR	Ejaz and von Korff (2017)
		AGT GGT GCA GCT AGC ATT TGA GAC		
HvADP	***AJ508228.2***	GCT CTC CAA CAA CAT TGC CAA C	qRT-PCR	unpublished
		GAG ACA TCC AGC ATC ATT CAT TCC		
Ppd-H1	***AY970701***	GAT GGA TTC AAA GGC AAG GA	qRT-PCR	Campoli et al. (2012b)
		GAA CAA TTG GCT CCT CCA AA		
HvFT1	***DQ100327***	GGT AGA CCC AGA TGC TCC AA	qRT-PCR	Campoli et al. (2012a)
		TCG TAG CAC ATC ACC TCC TG		
HvFT2	***DQ297407.1***	TAC CGA GGT TGT GTG CTA CG	qRT-PCR	Digel et al. (2015)
		TCACATCCTTCTCCCGCCGG		
HvFT3	***ADW83186.1***	TGT CAG ACA TCC CTG GAA CA	qRT-PCR	unpublished
		CTG ATC CAC CTT CCC TTT GA		
HvFT4	***DQ411320.1***	AAA CGA TAG CTT TGG AAA AGA GAT C	qRT-PCR	unpublished
		TCC TCC CGC CGG TGC CCG TG		
HvBM1	***AJ249142.1***	AGA GGA GAA CGC AAG GCT AAA GG	qRT-PCR	Trevaskis et al. (2007)
		AGT TGA AGA GTG ATA ATC CGA GCC TGA G		
HvBM3	***AJ249143***	GCC GTC ACC AGC ACA AGC AA	qRT-PCR	Digel et al. (2015)
		CCC CAT TCA CCC TGT AGC AAA GA		
VRN-H1	***AY750995***	CTG AAG GCG AAG GTT GAG AC	qRT-PCR	Campoli et al. (2012a)
		TTC TCC TCC TGC AGT GAC CT		
HvBM8	***AJ249146***	CCA CAG CAG CCG ACA CCT A	qRT-PCR	Digel et al. (2015)
		TGC CTT TGG GGG AGA AGA CG		
HvBM10	***EF043040.1***	GCT CAT CGT CTT CTC CTC CAC	qRT-PCR	Trevaskis et al. (2007)
		CTC CTC GCC TCT CAT CTG TC		
INT-C	***KY070602.1***	TTT GAT CAA TCG CTC CTC GT	qRT-PCR	unpublished
		GAG TTG GCA AAC ACC ACT CC		

Supplementary Table 2: FT4 orthologs in the genomes of 24 monocot species.

**Species**	**Ensemble accession**	**Alignment gene ID**
Aegilops tauschii	AET2Gv20263500.1	AtFT4
Brachypodium distachyon	KQK18033	BdFT4
Eragrostis curvula	TVU07845	EcFT4
Eragrostis tef	Et_s1327-4.40-1.mrna1	EtFT4_a
Eragrostis tef	Et_s9869-0.28-1.mrna1	EtFT4_b
Hordeum vulgare	HORVU2Hr1G023180.1	HvFT4
Leersia perrieri	LPERR06G14970.1	LpFT4
Oryza barthii	OBART06G18170.1	ObFT4
Oryza brachyantha	OB06G25800.1	ObrFT4
Oryza glaberrima	ORGLA06G0151500.1	OgFT4
Oryza glumipatula	OGLUM06G19250.3	OgluFT4
Oryza longistaminata	KN539494.1_FGT006	OlFT4
Oryza meridionalis	OMERI06G18710.1	OmFT4
Oryza punctata	OPUNC06G16320.1	OpFT4
Oryza rufipogon	ORUFI06G19280.1	OrFT4
Oryza sativa	Os06t0552900-00	OsFT4
Panicum hallii	PAN23758	PhFT4
Saccharum spontaneum	Sspon.08G0007600-2B-mRNA-1	SsFT4_a
Saccharum spontaneum	Sspon.08G0007600-1A-mRNA-1	SsFT4_b
Saccharum spontaneum	Sspon.08G0007600-3C-mRNA-1	SsFT4_c
Setaria italica	KQL10977	SiFT4
Sorghum bicolor	EER89918	SbFT4
Triticum aestivum	TraesCS2A02G132300.1	TaFT4_A1
Triticum aestivum	TraesCS2B02G154800.1	TaFT4_B1
Triticum aestivum	TraesCS2D02G134200.1	TaFT4_D1
Triticum aestivum	TraesCS7B02G272600.1	TaFT4_A2
Triticum dicoccoides	TRIDC2AG016280.2	TgFT4_a
Triticum dicoccoides	TRIDC2BG019610.2	TgFT4_b
Triticum dicoccoides	TRIDC7BG044260.1	TgFT4_c
Triticum turgidum	TRITD2Bv1G048450.1	TtFT4
Triticum urartu	TRIUR3_23989-T1	TuFT4
Zea mays	Zm00001d046300_P001	ZmFT4

Supplementary Table 3: Protein IDs or gene model information used for the multiple sequence alignment in Figure 6. TAIR and GenBank were searched on 21.11.2017.

Protein name	Protein ID / Gene Model	HORVU.MOREX.r2	Species	Function	Protein sequence retrieved from
AtFT	AT1G65480.1		Arabidopsis thaliana	Induction	TAIR
AtTFL1	AT5G03840.1		Arabidopsis thaliana	Repression	TAIR
AcFT1	AGZ20207.1		Allium cepa (onion)	Induction	GenBank
AcFT4	AGZ20210.1		Allium cepa (onion)	Repression	GenBank
BvFT1	ADM92608.1		Beta vulgaris (sugar beet)	Repression	GenBank
BvFT2	ADM92610.1		Beta vulgaris (sugar beet)	Induction	GenBank
DlFT1	AEZ63949.1		Dimocarpus longan (longan)	Induction	GenBank
DlFT2	AEZ63950.1		Dimocarpus longan (longan)	Repression	GenBank
GmFT2a	BAJ33491.1		Glycine max (soybean)	Induction	GenBank
GmFT4	NP_001276224.1		Glycine max (soybean)	Repression	GenBank
GmFT5a	BAJ33494.1		Glycine max (soybean)	Induction	GenBank
HaFT1	ADF32943.1		Helianthus annuus (sunflower)	Repression	GenBank
HaFT4	ADF32945.1		Helianthus annuus (sunflower)	Induction	GenBank
NtFT1	AFS17369.1		Nicotiana tabacum (tobacco)	Repression	GenBank
NtFT2	AFS17370.1		Nicotiana tabacum (tobacco)	Repression	GenBank
NtFT3	AFS17371.1		Nicotiana tabacum (tobacco)	Repression	GenBank
NtFT4	AFS17372.1		Nicotiana tabacum (tobacco)	Induction	GenBank
PaFTL1	AEH59567.1		Picea abies (norway spruce)	Repression	GenBank
PaFTL2	ABQ85553.1		Picea abies (norway spruce)	Repression	GenBank
ScFT1	AHZ46121.1		Saccharum ssp. (sugarcane)	Repression	GenBank
TaFT1_A1	Traes_7AS_EBD5F1F54.1		Triticum aestivum (wheat)	Induction	Halliwell et al., 2016
OsFTL2 (Hd3a)	Os06g06320.1		Oryza sativa (rice)	Induction	Halliwell et al., 2016
OsFTL3	Os06g06300.1		Oryza sativa (rice)	Induction	Halliwell et al., 2016
OsFTL1-in	Os01g11940.1		Oryza sativa (rice)	Induction	Halliwell et al., 2016
HvFT1	DQ100327	HORVU.MOREX.r2.7HG0542540.1	Hordeum vulgare (barley)	Induction	Halliwell et al., 2016
HvFT2	DQ297407	HORVU.MOREX.r2.3HG0203250.1	Hordeum vulgare (barley)	Induction	Halliwell et al., 2016
HvFT3	HM133572	HORVU.MOREX.r2.1HG0062650.1	Hordeum vulgare (barley)	Induction	Halliwell et al., 2016
HvFT4	DQ411320.1	HORVU.MOREX.r2.2HG0096390.1	Hordeum vulgare (barley)	Repression	Halliwell et al., 2016

**Protein name**	**Protein ID / Gene Model**	**HORVU.MOREX.r2**	**Species**	**Function**	**Protein sequence retrieved from**
HvFT5	EF012202.1	HORVU.MOREX.r2.4HG0348940.1	Hordeum vulgare (barley)	unknown	Halliwell et al., 2016
HvFT6	Morex_contig_54196	HORVU.MOREX.r2.6HG0474950.1	Hordeum vulgare (barley)	unknown	Halliwell et al., 2016
HvFT7	Morex_contig_1573409	HORVU.MOREX.r2.5HG0385730.1	Hordeum vulgare (barley)	unknown	Halliwell et al., 2016
HvFT8A	Morex_contig_37453	HORVU.MOREX.r2.2HG0173130.1	Hordeum vulgare (barley)	unknown	Halliwell et al., 2016
HvFT8B	Morex_contig_1560712	n.d.	Hordeum vulgare (barley)	unknown	Halliwell et al., 2016
HvFT8C	Morex_contig_158449	HORVU.MOREX.r2.2HG0165870.1	Hordeum vulgare (barley)	unknown	Halliwell et al., 2016
HvFT9	MLOC_58552.3	HORVU.MOREX.r2.2HG0146670.1	Hordeum vulgare (barley)	unknown	Halliwell et al., 2016
HvFT10	Morex_contig_44860	n.d.	Hordeum vulgare (barley)	unknown	Halliwell et al., 2016
HvFT11	MLOC_57326.1	HORVU.MOREX.r2.4HG0285050.1	Hordeum vulgare (barley)	unknown	Halliwell et al., 2016
HvFT12	MLOC_64619.2	n.d.	Hordeum vulgare (barley)	unknown	Halliwell et al., 2016

Supplementary Table 4: Significant differences for temporal and developmental expression levels of flowering time genes in the leaves of Ubi::HvFT4, null segregant and Golden Promise plants (Figure 4).

Gene	Time point or developmental phase	Golden Promise	Null segregant	Ubi::HvFT4-517	Significance code
**HvFT4**	21 DAE	a	a	b	**
	29 DAE	a	a	b	**
	35 DAE	a	a	b	**
	42 DAE	a	NA	b	*
	49 DAE	a	a	b	**
	57 DAE	a	a	b	**
	64 DAE	a	a	b	**
	Spikelet initiation	a	a	b	**
	Early reproductive growth	a	a	b	**
	Late reproductive growth	a	a	b	**
	Flowering	a	a	b	**
**HvFT1**	21 DAE	a	a	a	
	29 DAE	a	a	a	
	35 DAE	a	a	a	
	42 DAE	a	NA	a	
	49 DAE	a	a	a	
	57 DAE	a	a	a	
	64 DAE	ab	b	a	*
	Spikelet initiation	a	a	a	
	Early reproductive growth	a	a	a	
	Late reproductive growth	a	a	a	
	Flowering	a	a	a	
**HvFT2**	21 DAE	n.d.	n.d.	n.d.	
	29 DAE	n.d.	n.d.	n.d.	
	35 DAE	n.d.	n.d.	n.d.	
	42 DAE	a	NA	n.d.	*
	49 DAE	a	b	n.d.	**
	57 DAE	a	a	a	
	64 DAE	ab	a	b	*
	Spikelet initiation	a	a	a	
	Early reproductive growth	a	a	a	
	Late reproductive growth	a	a	a	
	Flowering	a	a	a	
**HvFT3**	21 DAE	a	a	a	
	29 DAE	a	a	a	
	35 DAE	a	a	a	
	42 DAE	a	NA	a	
	49 DAE	a	b	a	*
	57 DAE	a	a	a	
	64 DAE	a	a	a	
	Spikelet initiation	a	a	a	
	Early reproductive growth	a	a	a	
	Late reproductive growth	a	a	a	

Gene	Time point or developmental phase	Golden Promise	Null segregant	Ubi::HvFT4-517	Significance code
	Flowering	a	a	a	
**Ppd-H1**	21 DAE	a	a	a	
	29 DAE	a	a	a	
	35 DAE	a	a	a	
	42 DAE	a	NA	a	
	49 DAE	a	a	a	
	57 DAE	a	a	a	
	64 DAE	a	a	a	
	Spikelet initiation	a	a	a	
	Early reproductive growth	a	a	a	
	Late reproductive growth	a	b	a	*
	Flowering	a	b	ab	
**VRN-H1**	21 DAE	a	a	b	**
	29 DAE	a	a	b	**
	35 DAE	a	b	c	**
	42 DAE	a	NA	b	*
	49 DAE	a	a	b	**
	57 DAE	a	a	b	**
	64 DAE	ab	a	b	*
	Spikelet initiation	a	ab	b	*
	Early reproductive growth	b	b	a	**
	Late reproductive growth	b	b	a	**
	Flowering	a	a	a	
**HvBM3**	21 DAE	ab	a	b	*
	29 DAE	a	a	a	
	35 DAE	a	a	b	**
	42 DAE	a	NA	b	*
	49 DAE	a	a	b	**
	57 DAE	a	a	b	**
	64 DAE	a	a	a	
	Spikelet initiation	a	a	a	
	Early reproductive growth	a	a	a	
	Late reproductive growth	a	a	a	
	Flowering	a	a	a	
**HvBM8**	21 DAE	n.d.	n.d.	n.d.	
	29 DAE	n.d.	n.d.	n.d.	
	35 DAE	n.d.	n.d.	n.d.	
	42 DAE	a	NA	n.d.	*
	49 DAE	ab	a	n.d.	**
	57 DAE	ab	a	b	*
	64 DAE	a	b	c	**
	Spikelet initiation	n.d.	n.d.	n.d.	
	Early reproductive growth	n.d.	n.d.	n.d.	
	Late reproductive growth	ab	b	a	*
	Flowering	a	b	a	*
**HvBM1**	21 DAE	a	a	a	
	29 DAE	a	a	a	

Gene	Time point or developmental phase	Golden Promise	Null segregant	Ubi::HvFT4-517	Significance code
	35 DAE	a	a	a	
	42 DAE	a	NA	b	*
	49 DAE	a	a	b	**
	57 DAE	a	ab	b	*
	64 DAE	a	a	a	
	Spikelet initiation	a	a	a	
	Early reproductive growth	a	a	a	
	Late reproductive growth	a	ab	b	*
	Flowering	a	a	a	
**HvBM10**	21 DAE	a	a	a	
	29 DAE	a	a	a	
	35 DAE	a	a	a	
	42 DAE	a	NA	a	
	49 DAE	a	a	b	**
	57 DAE	a	a	b	**
	64 DAE	a	a	a	
	Spikelet initiation	a	a	a	
	Early reproductive growth	a	a	a	
	Late reproductive growth	a	ab	b	*
	Flowering	a	a	a	

NA: Expression data are not available.

n.d.: Expression was below detection limit.

*: Ubi::HvFT4-517 differs significantly from one control line (Golden Promise or null segregant),

**: Ubi::HvFT4-517 differs significantly from both control lines (Golden Promise and null segregant).

Statistical differences ($p \leq 0.05$) between genotypes where determined by one-way analysis of variance (one-way ANOVA) followed by Tukey's multiple comparison test (Tukey HSD). DAE: days after emergence.

Supplementary Figures:

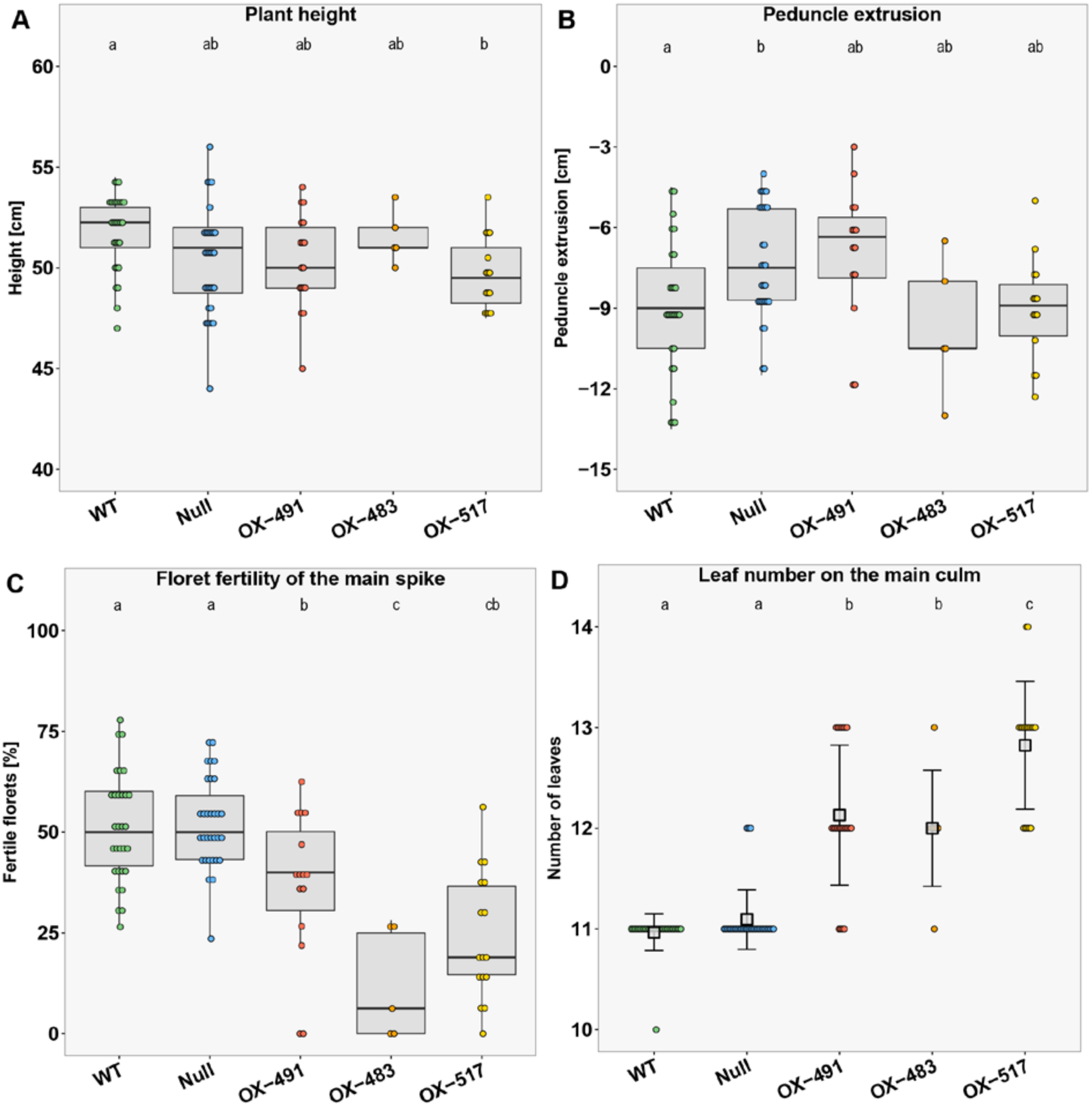

Supplementary Figure 1: Overexpression of *HvFT4* decreases tillering and increases the number of leaves on the main shoot, but does not influence plant height and peduncle extrusion. Morphological phenotypes were measured at heading **A:** Plant height, **B**: Peduncle extrusion, **C:** floret fertility, **D:** number of leaves on the main culm. The grey boxes in plot D indicate the mean ± SD). Each dot represents the values obtained from a single plant. Statistical differences ($p \leq 0.05$) between families were calculated by one-way analysis of variance (one-way ANOVA) followed by Tukey's multiple comparison test (Tukey HSD). **WT** = Golden Promise, **Null** = null segregant, **OX-491** = *Ubi::HvFT4*-491, **OX-483** = *Ubi::HvFT4*-483, **OX-517** = *Ubi::HvFT4*-517.

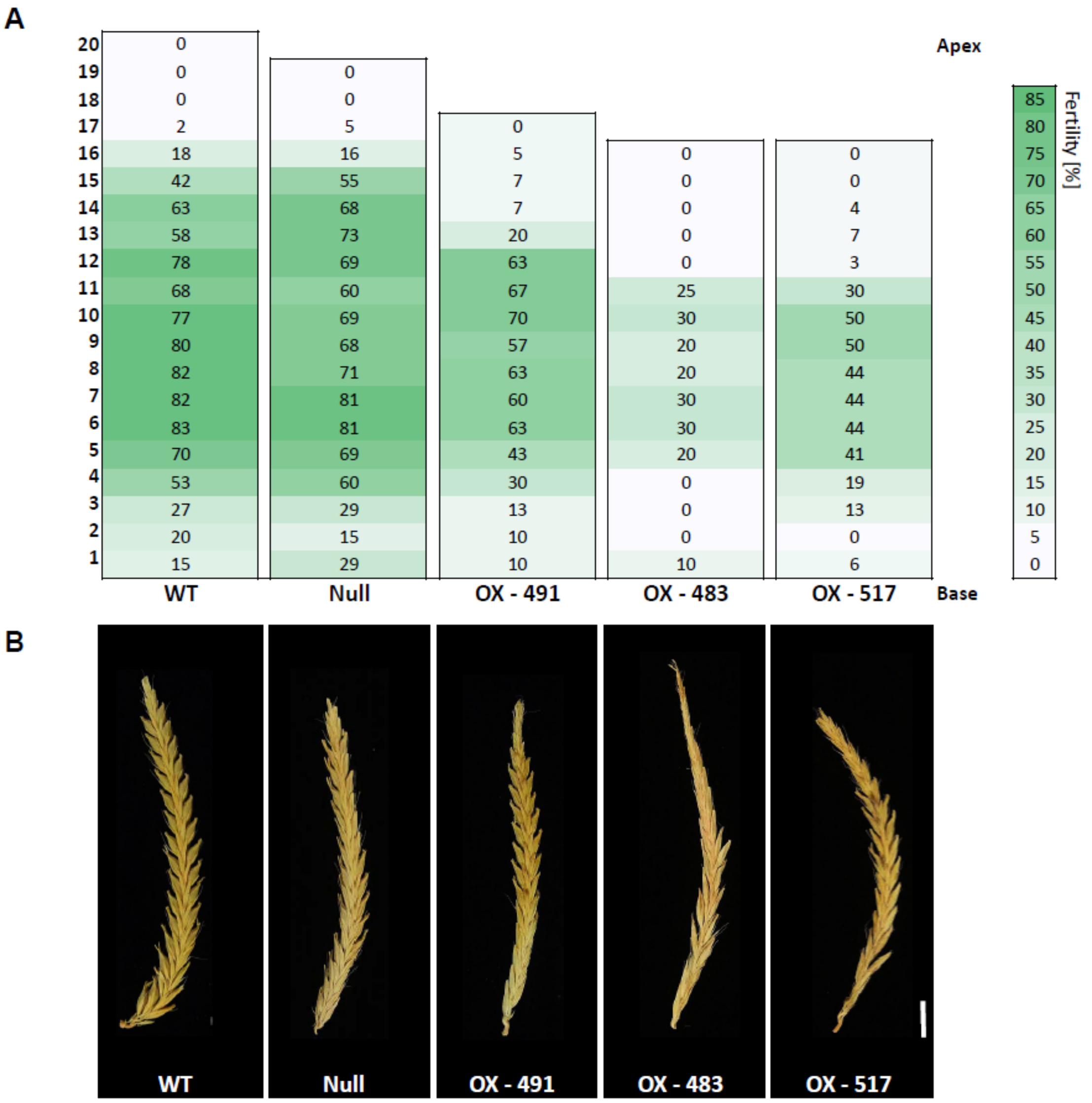

Supplementary Figure 2: Overexpression of *HvFT4* reduces floret fertility of the main shoot spike. A: Floret fertility of the main shoot spike. The color intensity represents the average fertility of the central floret on every rachis node (node 1 to 20) of the main spikes (cf. **Error! Reference source not found.** C). Numbers (0 - 85) indicate the respective average floret fertility in percent [%]. **B**: Representative main shoot spikes. Scale bar represents 1 cm. Awns were cut off for clear visualization. **WT** = Golden Promise, **Null** = null segregant, **OX-491** = *Ubi::HvFT4*-491, **OX-483** = *Ubi::HvFT4*-483, **OX-517** = *Ubi::HvFT4*-517.

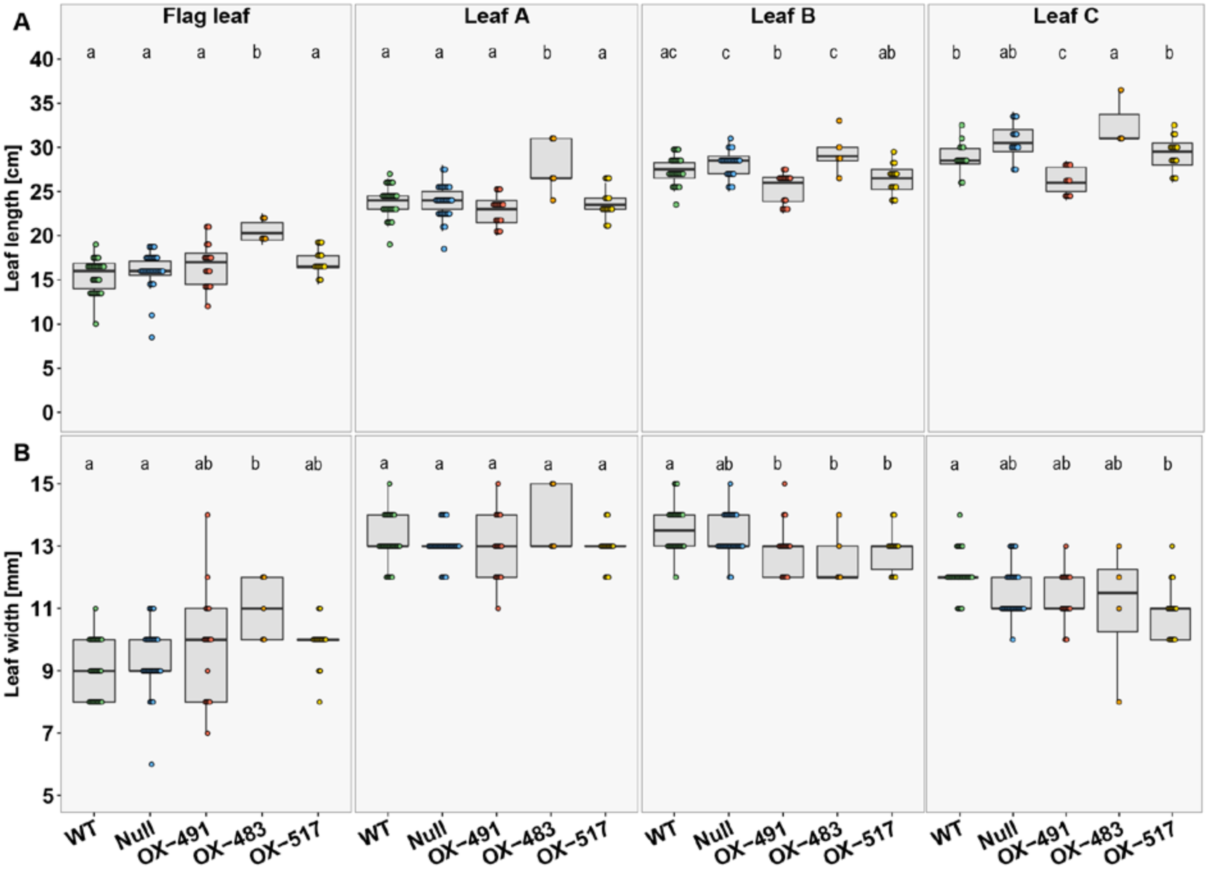

Supplementary Figure 3: Overexpression of *HvFT4* does not influence leaf size. A: Leaf length and **B:** leaf width of flag leaf and the three leaves below the flag leaf (leaf A, B, C) were measured on the main shoot at heading. Each dot represents the values obtained from a single plant. Statistical differences ($p \leq 0.05$) between families were calculated by one-way analysis of variance (one-way ANOVA) followed by Tukey's multiple comparison test (Tukey HSD). **WT** = Golden Promise, **Null** = null segregant, **OX-491** = *Ubi::HvFT4*-491**, OX-483** = *Ubi::HvFT4*-483, **OX-517** = *Ubi::HvFT4*-517.

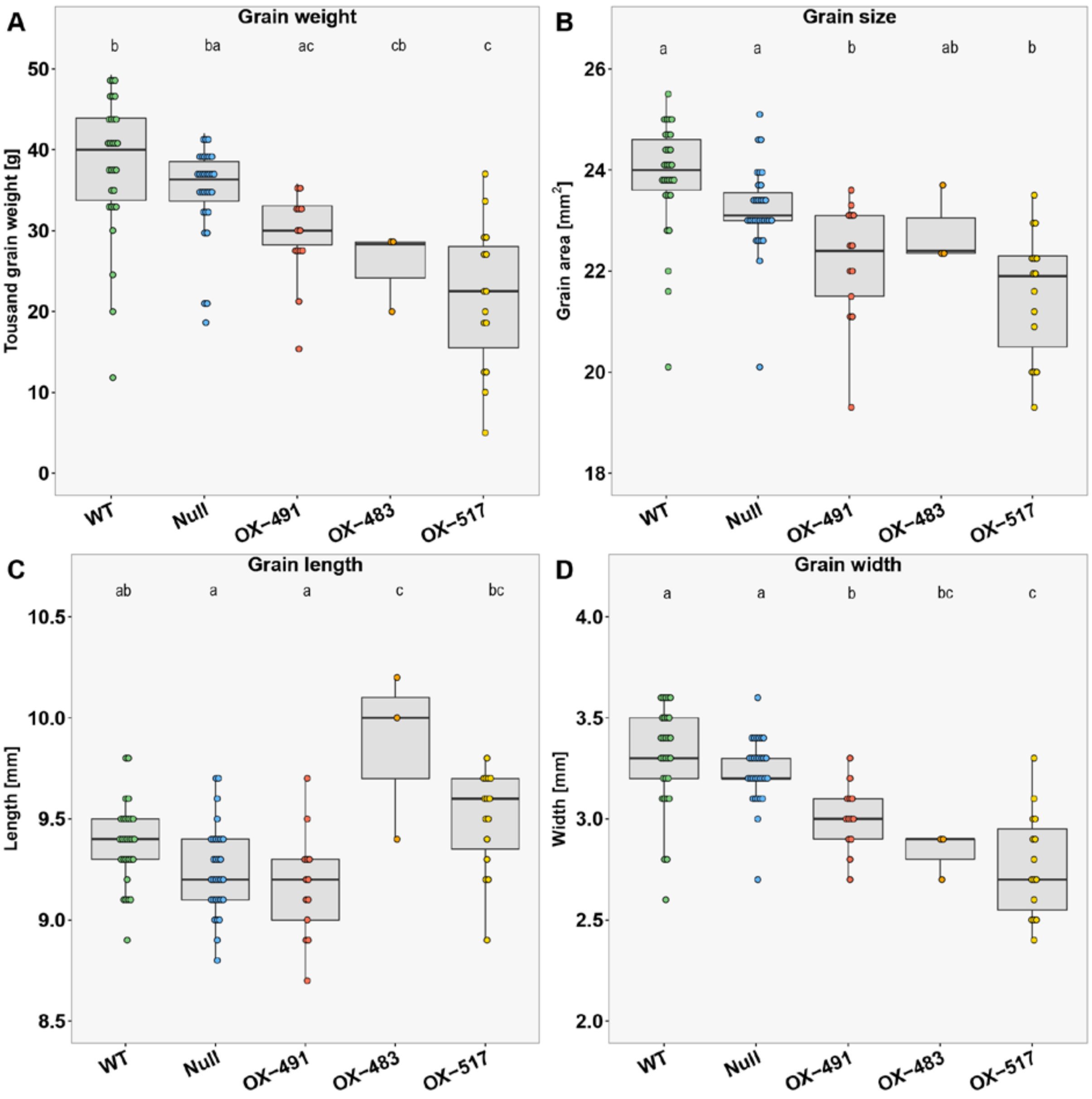

Supplementary Figure 4: Overexpression of *HvFT4* reduces seed weight and width. A: Seed weight, **B:** Seed size, **C:** Seed length and **D:** Seed width. Each dot represents the mean thousand grain weight, grain size, length or width of the seeds derived from the main shoot spike of a single plant. Statistical differences ($p \leq 0.05$) between genotypes were calculated by one-way analysis of variance (one-way ANOVA) followed by Tukey's multiple comparison test (Tukey HSD). **WT** = Golden Promise, **Null** = null segregant, **OX-491** = *Ubi::HvFT4*-491, **OX-483** = *Ubi::HvFT4*-483, **OX-517** = *Ubi::HvFT4*-517.

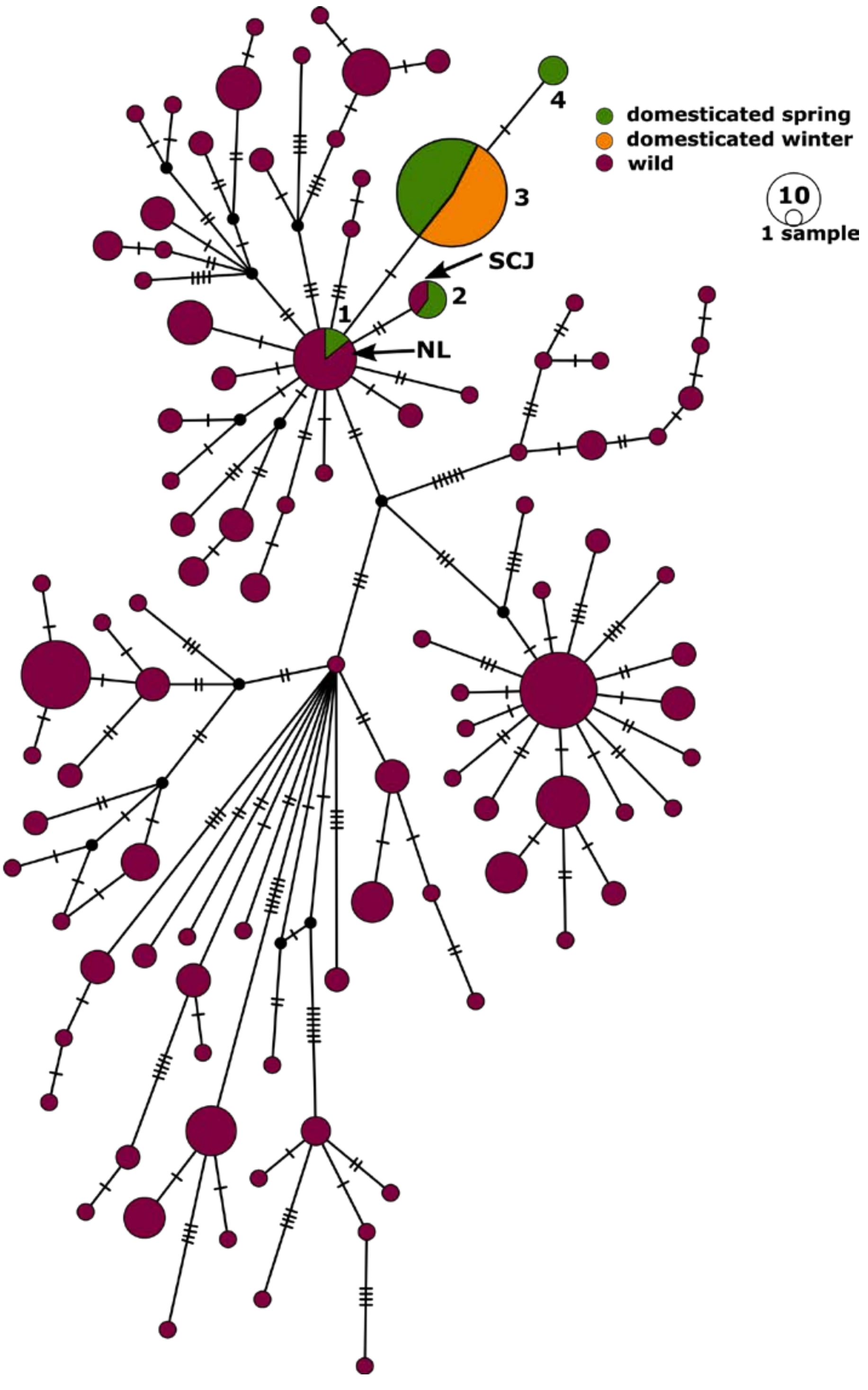

Supplementary Figure 5: A median joining haplotype network of 96 *HvFT4* haplotypes. The *HvFT4* haplotypes were identified from the target enrichment data of 248 wild (dark red) and 51 winter (orange) and spring (green) domesticated barley accessions (Pankin *et al.*, 2018). The size of the circles corresponds to the number of barley genotypes carrying each of the haplotypes. The numbers (1-4) denote the cultivated barley *HvFT4* haplotypes. NL (North Levant) and SCJ (Sharon, Coastal Plain and Judean Lowlands) indicate wild barley populations (*sensu* Pankin et al., 2018) carrying *HvFT4* haplotypes shared with the cultivated barley.

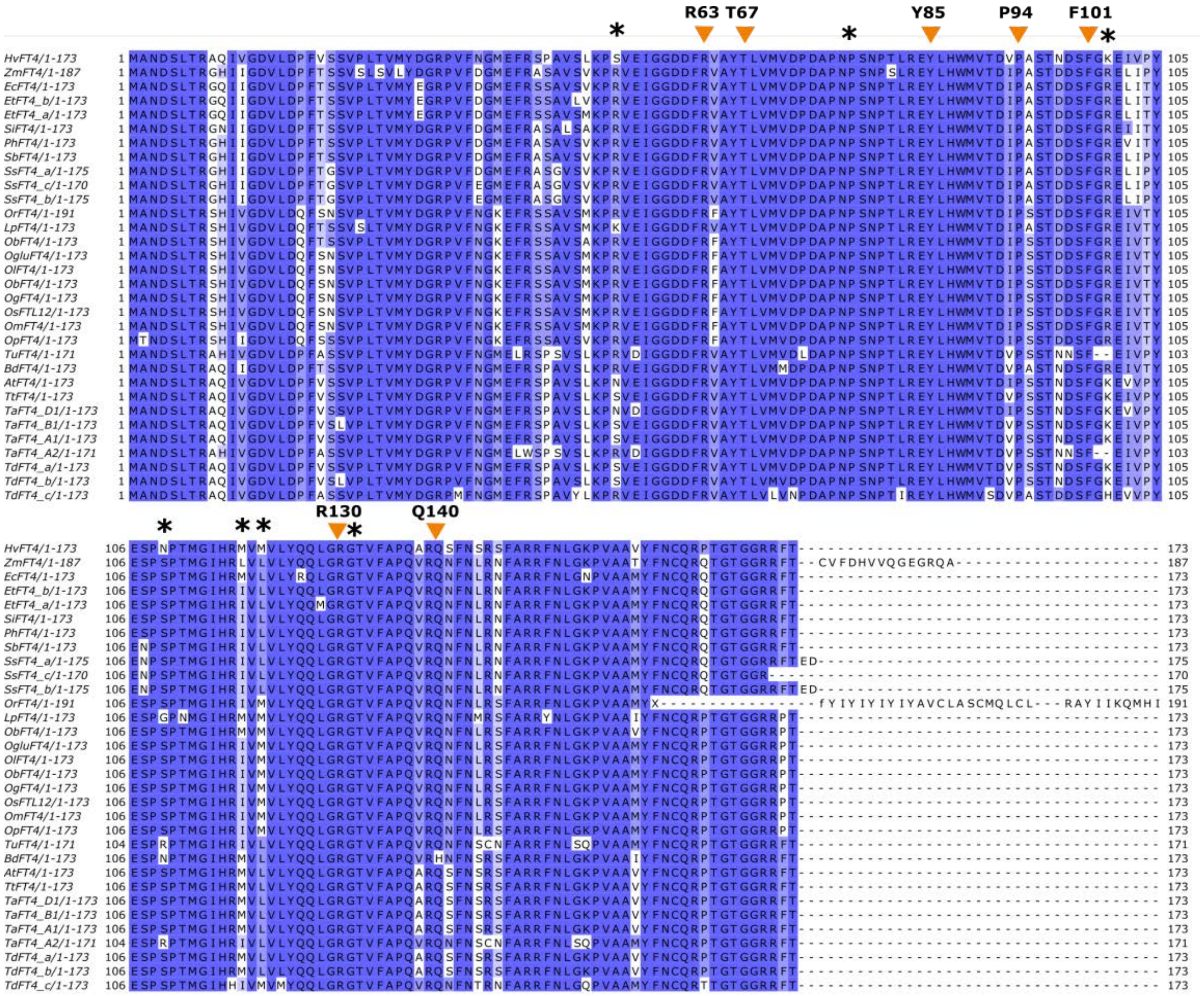

Supplementary Figure 6. Multiple alignment of the FT4 orthologs from 24 monocot grass species. The shades of blue illustrate conservation of the amino acid (aa) residues. The aa residues putatively critical for the FT4 function are shown by red triangles. The sequence names are as in Supplementary Table 2. The length of the protein is separated from the sequence name by a forward slash. Asterisks indicate the position of non-synonymous substitutions identified in the diverse panel of wild and cultivated barley (Supplementary Figure 5).
